# Barriers and Facilitators to Health Care AI Adoption Among Those Living in Wales and Working in Health Care in Wales: Online Survey

**DOI:** 10.2196/81543

**Published:** 2025-12-05

**Authors:** Michal Pruski, Katherine E Woolley, Kathleen L Withers

**Affiliations:** 1 CEDAR Cardiff and Vale University Health Board Cardiff United Kingdom; 2 School of Health Science University of Manchester Manchester United Kingdom; 3 School of Engineering Cardiff University Cardiff United Kingdom; 4 School of Medicine Cardiff University Cardiff United Kingdom

**Keywords:** artificial intelligence, surveys and questionnaires, trust, public attitudes, consumer health informatics

## Abstract

**Background:**

NHS Wales routinely collects patient-reported outcome measures, and these, together with other clinical data, offer an opportunity to design machine learning (ML) technologies that could advance the implementation of prudent health care principles (a health care strategy encouraged by the Welsh Government). However, the wide adoption of such technologies is not only dependent on the development of technically well-performing ML algorithms but also on end-user barriers and facilitators.

**Objective:**

This study aimed to identify potential end-user (patient and health care professional) barriers and facilitators to the use of ML in health care decision-making in Wales. The study’s objective was to provide actionable information for those who are developing and implementing ML technologies in health care, rather than contributing to the theoretical advance of technology implementation frameworks.

**Methods:**

An online survey using Microsoft Forms was conducted. It was open to anyone who was 16 years or older and lived in Wales (member of the public criterion) or was a registered health care professional working in Wales and participating in treatment or therapy decision-making (health care professional criterion). The anonymous survey was open from December 4, 2024, to March 4, 2025. The survey used single-choice, ranking, and free-text questions, which were phrased differently for both eligibility groups. Data analysis was based on the respondent-selected eligibility criterion and self-declared general attitude toward health care artificial intelligence (AI; generally supportive, opposed, or uncertain), using descriptive and inferential statistics, as well as a summary of free-text responses.

**Results:**

A total of 309 respondents filled out the survey, 179 selecting the member of the public criterion and 130 selecting the health care professional criterion. Among them, 209 self-identified as having a generally supportive attitude toward health care AI, 31 as generally being opposed to health care AI, and 69 as being uncertain. Overall, respondents placed a large emphasis on the presence of evidence for the technology’s effectiveness and humans being in control of the health care process, even if this meant that care processes were not as fast as they could be with a higher degree of automation. Those with a negative attitude toward AI placed more emphasis on human autonomy than other respondent groups.

**Conclusions:**

Those developing and implementing health care AI technologies should develop an unbiased evidence base for the effectiveness of their technologies, using transparent methodologies, and continue their evaluation when the technology is in place. Moreover, implementation should not decrease patient-clinician contact but automate specific tasks only and maintain a human in the loop.

## Introduction

### Technology Adoption

Technology adoption within health care is particularly complex, as it involves contingent innovation-decisions [1], with technologies often requiring regulatory approval to permit the use of a technology, adoption of the technology by a specific organization, a decision to use the technology by a health care professional, and the consent to its use by the patient. Consequently, adoption decisions depend on technology, organizational, and personal factors [2]. Furthermore, diffusion of innovation research has identified 5 adopter categories based on how soon they adopt a technology [1], which are broadly likely to relate to “pro-AI” and “AI-cautious” groups identified in health informatics research [3].

A wide range of theoretical frameworks have been applied to aid and assess digital health care technology implementation, with reports varying as to which framework is the most popular [4,5]. While such frameworks use many distinct concepts, these concepts often interrelate across frameworks. For example, there is a clear overlap between concepts of “perceived ease of use” and “individual effort expectancy,” as well as “perceived usefulness” and “performance expectancy,” which are used respectively in the Technology Acceptance Model (TAM) and the Unified Theory of Acceptance and Use of Technology (UTAUT). Nevertheless, at the core of these frameworks is the ability to predict user acceptance [4].

### Importance of the End User Research in AI Adoption

A briefing by a leading UK health think-tank noted that artificial intelligence (AI) adoption within health care needs to be driven by the public [6]. Without end-user acceptance, theoretically useful digital solutions that fail to address user concerns and preferences might not realize their potential benefits. The briefing calls for the engagement of both patients and staff in the design and development of AI technologies [6].

Such an approach is consistent with current UK health care research guidelines, which emphasize public involvement throughout the research lifecycle (including the methodology development phase) and highlight that public involvement increases the likelihood of research results being more useful and beneficial to patients [7,8]. This is reflected in the National Institute for Health and Care Excellence’s (NICE) policy on public involvement in its work on clinical guidelines [9].

Importantly, many AI implementation questions cannot be answered from a purely technical perspective. For example, during the design or implementation of a machine learning (ML) technology, choices often need to be made to prioritize one aspect of the technology over another, such as when conflicts occur between privacy and efficacy, or efficacy and justice considerations [10,11]. While organizations might have preferences over how these are balanced, ultimately if patients or clinicians disagree with this balance, the technology might not be adopted in practice.

### NHS Wales Considerations

National Health Service (NHS) Wales consists of various organizations, which all use digital technologies. While some national solutions are provided by Digital Health and Care Wales (DHCW) and TEC Cymru, health boards and trusts often make their own choices related to technology adoption and also bear the consequences of these choices. As such, these organizations require information on their own populations to make these decisions effectively.

However, Wales also has an extensive nationwide program for collecting patient-reported outcome measures (PROM) data as part of routine clinical practice. A previous literature review has shown that there is potential for such data to be used together with ML techniques to help predict patients’ postintervention outcomes [12]. Such predictive health care ML technologies have the potential to provide information to patients and clinicians to help them make better-informed treatment and therapy decisions. Furthermore, ML technologies have the potential to aid the application of the prudent health care and value-based principles, which are encouraged in NHS Wales by the Welsh Government [13].

Importantly, while UK-wide studies have looked at barriers and facilitators to health care AI adoption, these have been dominated by respondents from England [14-16]. Since health care is a matter devolved to the Welsh Government, the NHS is organized and funded differently in Wales compared to England, and Wales has an older and more rural-based population than England; implementation of AI technologies in Wales is dependent on different geographical, organizational, and population factors [17,18]. The only large-scale study undertaken in Wales on the adoption of AI in health care was the 2025 “Time to Talk Public Health” survey, which had 2137 respondents and included a single question on user acceptability of AI in breast screening, and ran concurrently to the survey described in this study [19]. Nevertheless, that study did not look at specific barriers and facilitators to adoption nor the information assurances needed to make these clinical AI technologies acceptable to users, but only at attitudes toward and perceived impact of such use of AI.

### Research Questions and Study Contributions

The study’s primary research question was: “What are the barriers and facilitators to ML adoption in clinical decision-making among end users in Wales?”

The study’s secondary research question was: “Are there differences in barriers and facilitators to ML adoption between respondents with different attitudes toward AI?”

To our best of our knowledge, this is the first all-Wales study looking at barriers and facilitators to AI adoption. It identifies barriers and facilitators to health care AI adoption in 2 distinct user populations: members of the public (as potential patients) and health care professionals who play a role in the treatment or therapy decision process. These results will offer guidance to both national and regional organizations on how to develop and implement such technologies.

It is one of the few studies that compares the responses between groups with different attitudes toward AI. As such, it contributes toward a better understanding of how to make such technologies more acceptable to AI skeptics.

The study questionnaire was designed with a focus on ML applications using PROM data to enable potential future benefits from the operationalization of such data within the therapeutic context. Nevertheless, we hope that the results of this study might be of use to a wider range of health care AI applications. While it was not an explicit aim of this study, it might also contribute to the broader literature on theories of technology adoption.

Compared to other studies, the design of this research, while supported by a literature review, was largely driven by stakeholder input to ensure that it met the needs of the public and health care leaders. Additionally, this study used ranking technology methodology over rating methodology to provide more actionable information for those wanting to use these findings in practice.

## Methods

### Literature Search and Review

A search strategy (Multimedia Appendix 1) was developed and run in MEDLINE ALL (Ovid) to identify relevant records. The search was also adapted and run in the following 5 databases: Embase (Ovid), The Cochrane Library, Scopus, IEEE Xplore, and ACM Digital Library. The searches were carried out on October 11, 2023. Records were imported into EndNote 20 and deduplicated. Two reviewers (MP and KEW) independently screened studies at title and abstract and full-text using EndNote. One reviewer (MP) assessed all records at title and abstract against the inclusion criteria (Multimedia Appendix 1). Full texts were obtained and assessed by one reviewer (MP) against the inclusion criteria. At both stages, a second reviewer (KEW) checked all included records and 10% of excluded records, noting any discrepancies. Discrepancies were resolved through discussion. The searches retrieved 2044 records, with 1314 records remaining after deduplication. Following title and abstract screening, 134 records were assessed at full-text assessment. Of these, 74 records met the inclusion criteria and pertained to 74 studies. The reasons for the exclusion of the remaining 60 records are provided in Multimedia Appendix 1. The included studies were reviewed by a single reviewer (MP) who extracted the barriers and facilitators identified in each publication, which were then grouped into themes. There were 22 themes, found across 67 publications, pertaining to staff, while 13 themes, found among 19 publications, related to patients (Multimedia Appendix 1). Performance of the AI technology was the most prominent theme in both the staff and patient groups (Multimedia Appendix 1); performance covers such aspects as algorithmic accuracy and practical usefulness.

### Questionnaire Creation

Using the themes identified in the literature, together with a review of ethics principles in health care adoption and specific topic explorations undertaken as part of this project, initial sets of questions were developed [11,20-22].

Similar to the wider literature on barriers and facilitators to the adoption of AI in health care, the questionnaire was designed with a large focus on ethical considerations [23]. Feedback was then sought on the draft question set from the patient and public involvement (PPI) group, stakeholder groups, and organizational colleagues with experience in research, survey design, or those who might be potential survey responders. After refinement, final feedback was sought from the Cardiff and Vale University Health Board’s Patient Experience Team. Once the English version of the questionnaire was approved, the questionnaire was translated into Welsh.

To ensure that the findings from the study can be practicably applied, the questionnaire primarily used ranking and choice questions, as opposed to rating questions, even though rating questions have been commonly used in similar studies [24,25]. While there is an ongoing debate surrounding the appropriateness of rating and ranking questions, with both having their advantages, the use of both methods tends to result in a similar order of item importance [26-28]. Nevertheless, using rating questions can result in more actionable information, as it avoids ties and nondifferentiation between responses, though this can create bias when respondents genuinely do not have a preference [26]. Ranking questions had the “shuffle” option enabled to reduce the chances of influence of the order in which the options were presented on participant ranking decisions. All questions were set as “required,” except the final questions, which asked for participants' contact preferences if they wanted to participate in the further qualitative part of the study.

The initial draft consisted of 21 questions, including 4 demographic questions and 2 questions about the respondents’ general attitude toward AI based on a past report suggesting that this is a potentially useful way of clustering responders [3]. Some questions differed between the two eligibility criteria groups. Because of the limitations of Microsoft Forms, we had to ensure that each ranking option had no more than 10 options, but PPI and steering group feedback indicated that we should ideally not have more than 5 options for each question. For the questions that focused on the prioritization of ethical principles, we used the set of principles identified by Jobin et al [29], but discarded “trust” to narrow it to 10 principles. Trust was discarded, as stakeholders felt that user trust would be the general outcome of the correct application of the other 10 ethical principles. For other ranking questions, the list of potential options was narrowed down to 5 options, in the most extreme case, from a pool of 46 options, based on PPI and steering group feedback. In some cases, the reduction was achieved by selecting a statement of interest to the PPI and steering group, as well as amalgamating specific statements into more generic statements. The inclusion and exclusion of other questions were also guided by PPI and steering group feedback. To focus respondents on the use of ML in the context of prudent health care, a vignette outlining a hypothetical use of ML with PROM data was presented in the questionnaire’s introduction section and then again after the demographic question section. The questionnaire was kept as short as possible, while keeping it informative, to increase the chances of questionnaire completion and be considerate of participant privacy.

The questionnaire was divided into 2 arms so that questions could be phrased in a way that was more relevant to members of the public in one arm and the health care professionals in the other arm. The questionnaire was anonymous, except when participants chose to leave their details for consideration in a further qualitative part of the project. The question inquiring about the respondents’ ethnicity was taken from the standard recommended set for Wales by the Office for National Statistics (ONS), and the age categories were adapted from ONS 6a and 6f categories to account for our target population [30-32].

The final English version of the questionnaire can be found in Multimedia Appendix 2. This was also translated into Welsh, and both versions of the questionnaire were available on the same link.

### Public and Professional Steering Group

The project had extensive PPI as well as input from a wider steering group. Two lay members sat on the PPI group and provided regular input into shaping the project and making sure the language used in the survey was appropriate and accessible. Moreover, a volunteer editorial panel from the Cardiff and Vale University Health Board’s Patient Experience Team also proofread the survey and provided comments. The project also benefited from the input of the project’s steering group, which consisted of a diverse range of professionals from the NHS, academia, and government. Additionally, the project team consulted other members of their department and staff experienced in survey design, working at the University Hospital of Wales, regarding various aspects of the questionnaire wording.

### Ethical Considerations

The project’s documentation was first submitted to Cardiff and Vale University Health Board’s research and development department (protocol number 8870) and subsequently submitted to the national Integrated Research Application System (IRAS ID 345131). The project was approved by Health and Care Research Wales (24/HCRW/0021) but was deemed exempt from needing approval by a research ethics committee. Implied consent was used, as the voluntary questionnaire was anonymous, and all participants had to acknowledge the privacy statement before being allowed to proceed with the questionnaire (Multimedia Appendix 2). To maintain privacy, only members of the research team had access to the full dataset, and all outputs were assessed for statistical disclosure. Participants received no compensation for taking part in the survey.

### Participants

The study used an open survey and convenience sampling. To be eligible to participate in the study, potential participants needed to have met at least one of two eligibility criteria: (1) members of the public living in Wales who were 16 years or older or (2) registered health care professionals working in Wales who make treatment or therapy decisions together with or for patients.

Members of the public living in Wales are all potential patients of health care services delivered in Wales, and therefore, the study was not limited to those who were current patients. The lower age limit of 16 years was selected as, in general, those who are 16 years old are considered to have the capacity to consent for health care treatment in the United Kingdom.

The second eligibility criterion was designed to allow those health care professionals who work in Wales but commute from England to also participate in the study. Because the study was a part of a wider research program focusing on improving value in health care and focused on the decision-making aspect of health care delivery, this criterion was limited to registered health care staff who participate in such decision-making.

Respondents could only select one eligibility criterion, even if they met both.

### Questionnaire Deployment

The questionnaire was deployed via the Microsoft Forms platform and hosted within the Cardiff and Vale University Health Board’s digital ecosystem, with the survey being voluntary and respondents not needing to fill it out to access any other part of the organization’s website. The links and QR codes to the questionnaire were distributed via social media, professional fora, messaging systems, and word of mouth. An advert was placed on our organizational website, and posters were placed throughout our organization. Advertising was undertaken in both English and Welsh; on social media, it was also disseminated in Polish. Key organizations and individuals were contacted to ask to distribute information about the study, including health care leaders, professional organizations, educational institutions, charities, religious and minority organizations, local government, governmental organizations, and members of the Senedd. The questionnaire was open from noon on December 4, 2024, to noon on March 4, 2025.

Respondents did not receive any incentives to fill out the survey. Participants could not change their answers after the questionnaire was submitted but could revisit the questions before submission. The system did not record site visits or uncompleted questionnaire attempts and did not use cookies or monitor IP addresses to identify potential duplicate entries. Timestamps were not monitored for atypical responses.

### Data Analysis

Data from the survey were analyzed in Microsoft Excel using descriptive statistics, with statistical hypothesis testing undertaken in R (version 4.1.3; R Foundation for Statistical Computing) using chi-square with or without Monte Carlo simulated *P* values (*B*=2000) depending on the frequencies in the contingency tables and post hoc assessment for the chi-square test (Bonferroni method, with or without Monte Carlo simulated *P* values, *B*=2000). For comparisons with demographic comparators, the tests were done on percentages rather than counts, as counts were not available for the reference population. For demographic data, “prefer not to say” data were excluded from the analysis. For all nondemographic questions, a comparison was made only between those with either a positive or negative self-declared attitude toward AI, following the general advice to reduce the degrees of freedom for post hoc analysis [33]. No inferential statistics were undertaken on the ranking results as ranks are not independent of each other [26]. A *P* value of <.05 was considered statistically significant. Respondent demographics were compared with those of the population of Wales and the NHS Wales workforce, as no statistics were available for the whole health care workforce for Wales. While the questionnaire asked respondents to choose their ethnicity using 18 groups listed by the ONS [31], results were presented using 5 overarching ONS groups to provide a more accessible overview of the results [31], and because StatsWales only provided this granularity of data [34]. Similarly, while participants were asked to state the county in which they lived or worked, these results have been amalgamated to the territories covered by the 7 NHS Wales health boards for a more accessible overview of the results.

The main subgroup analysis was carried out based on participants' self-reported attitude toward health care AI (support, oppose, or uncertain). All participants were asked to list the 3 most important barriers and facilitators to the adoption of such health care ML technologies, with this information collected via free-text fields. As such, themes had to be developed before statistical analysis could be performed on the answers to these questions. This step should not be confused with thematic analysis used in qualitative research but rather be seen as a method of accounting for potential variations in spelling, or because participants explained a concept rather than simply stating it.

To aid with the free-text analysis, the responses to these questions, segregated by eligibility criteria and attitude toward health care AI, were imported into MAXQDA 24 (VERBI Software). In MAXQDA 24, the AI Assist “Suggest Subcodes” function was used with the suggestion language “English” and the “add bullet list with examples for each subcode” option selected on March 28, 2025. The generated subthemes were compiled and reviewed by one researcher (MP), and sense-checked by another colleague based on MAXQDA 24's original suggestions. One researcher (MP) used this list as a starting point for coding the free-text responses, but adjusted the list by generating new subthemes or not using redundant MAXQDA 24–generated subthemes. After MP coded 100% of the free-text responses, 20% of the responses were checked by a second researcher (KEW). Discrepancies were resolved via discussion, and both researchers then agreed on the final themes. MAXQDA 24 AI Assist was not used to code individual responses.

The a priori analysis plan was to check how often specific barriers and facilitators were mentioned across all the questions, and how often specific barriers and facilitators were mentioned as the most important barrier or facilitator. If a respondent mentioned more than 3 items across the 3 free-text barrier questions and the 3 free-text facilitator questions, all of them would be included in the frequency count. If a respondent mentioned more than one item in the questions pertaining to the most important barrier and most important facilitator, it was a priori decided that the first item mentioned would be counted as the most important barrier or facilitator, unless the text indicated which of the mentioned items was to be considered as the most important.

Since participants had to complete all questions (except contact detail questions), no responses were discarded, even if only token answers were provided to the open questions. No statistical methods were used to adjust the answers for any nonrepresentative characteristics.

### Checklists

The CHERRIES (Checklist for Reporting Results of Internet E-Surveys) checklist has been used for this survey [35,36].

## Results

### Respondent Demographics

A total of 309 responses were collected. Respondent demographics are presented in Table 1, and additional health care staff demographics are presented in Multimedia Appendix 3. All respondents chose to fill out the questionnaire in English. The sex distribution did not differ significantly among the members of the public sample when compared to the reference populations (Table 1), but it did in the health care professional sample (Table 1). In the health care group, women were relatively underrepresented and men overrepresented (post hoc *P*=.04 for both sexes). Both ethnicity and age distribution did not differ significantly from the reference populations for both members of the public and health care professionals (Table 1). Among members of the public (Table 1), there was an overrepresentation of respondents from the region covered by the Cardiff and Vale University Health Board (post hoc *P*<.001). There was no reliable comparator for health care professionals. At a more granular level, at least one response was received from members of the public living in each Welsh county, but not from health care staff working within each county. Most respondents self-reported as having, in general, a positive attitude toward health care AI (Table 1).

Those who selected the eligibility criterion of being registered health care professionals working in Wales and being involved in therapeutic or treatment decision-making came from all major professional groups, with most (32/130, 24.6%) describing themselves as doctors or dentists, followed by health care scientists (29/130, 22.3%) (Multimedia Appendix 3). The largest self-reported primary areas of work were “other secondary care” and “diagnostics.” The majority of respondents declared that their primary patient groups were adults.

**Table 1:** Respondent demographics.^a^

Characteristics		Public (n=179)					Health care professionals (n=130)			
		Values, n (%)	Reference, %	Chi-square (*df*)	*P* value	Values, n (%)		Reference, %	Chi-square (*df*)	*P* value
**Sex**				0.9 (1)	.34				5.8 (1)	.02
	Female	102 (57.0)	51.1			75 (57.7)^b^		76.6^b^		
	Male	71 (39.7)	48.9			51 (39.2)^b^		23.4^b^		
	Prefer not to say	6 (3.6)	N/A^c^			4 (3.1)		N/A		
**Ethnicity**				1.2 (N/A)	>.99				0.8 (N/A)	>.99
	White	161 (89.9)	93.9			119 (91.5)		79.7		
	Asian or Asian British	7 (4.0)	3.0			4 (3.1)		4.6		
	Black, African, Caribbean, or Black British	4 (2.3)	0.8			2 (1.5)		1.3		
	Mixed or multiple ethnic groups	3 (1.7)	1.6			1 (0.8)		1.0		
	Other ethnic groups	1 (0.6)	1.3			1 (0.8)		1.4		
	Prefer not to say, not stated, or missing data	3 (1.7)	N/A			3 (2.3)		12.0		
**Age (years)**				8.9 (5)	.11				4.8 (N/A)	.29
	16-24	25 (14.0)	14.0			5 (3.8)		6.9		
	25-34	42 (23.5)	14.8			30 (23.1)		23.8		
	35-49	41 (22.9)	21.1			62 (47.7)		34.9		
	50-64	44 (24.6)	24.6			29 (22.3)		32.5		
	65-74	19 (10.6)	13.8			2 (1.5)		2.0		
	75 or over	4 (2.2)	11.7			0 (0)		N/A		
	Prefer not to say	4 (2.2)	N/A			2 (1.5)		N/A		
**Region**				25.1 (N/A)	<.001				N/A	N/A
	Aneurin Bevan UHB^d^	28 (15.6)	18.8			6 (4.6)		N/A		
	Betsi Cadwaladr UHB	14 (7.8)	21.9			10 (7.7)		N/A		
	Cardiff and Vale UHB	83 (46.4)^b^	16.4^b^			85 (65.4)		N/A		
	Cwm Taf Morgannwg UHB	18 (10.1)	14.1			2 (1.5)		N/A		
	Hywel Dda UHB	10 (5.6)	12.3			4 (3.1)		N/A		
	Powys THB^e^	4 (2.2)	4.2			3 (2.3)		N/A		
	Swansea Bay UHB	22 (12.3)	12.3			17 (13.1)		N/A		
	Prefer not to say	0 (0)	N/A			3 (2.3)		N/A		
**General health care AI^f^ attitude**				N/A	N/A				N/A	N/A
	Support	116 (65)	N/A			93 (72)		N/A		
	Oppose	23 (13)	N/A			8 (6.0)		N/A		
	Uncertain	40 (22)	N/A			29 (22)		N/A		

^a^Comparators for sex were from the Office for National Statistics (ONS) 2021 Census and StatsWales September 30, 2023, data; note that StatsWales presented the categories as “men” and “women” [37,38]. Comparators for ethnicity were taken from the Welsh Government’s website report on the 2021 Census and StatsWales September 30, 2023, data [39,40]. Age comparators were taken from the ONS 2021 Census and StatsWales September 30, 2023, data; note that the ONS data only allowed for the calculation of a 15-24 category rather than 16-24, and the StatsWales data only allowed for the following comparator categories to be calculated: ≤25, then 26-35, 36-50, 51-65, and >65 [37,41]. The only comparator used for region data was the StatsWales population estimate by local authority for midyear 2023 [42]. For post hoc test *P* values, see text.

^b^Statistically significant in post hoc analysis (see text for details).

^c^N/A: not applicable.

^d^UHB: University Health Board.

^e^THB: Teaching Health Board.

^f^AI: artificial intelligence.

### Demographics by AI Attitude

There were no statistically significant differences between those with a positive and negative attitude toward AI when comparing sex (Multimedia Appendix 3; *χ*^2^_1_=0.4, *P*=.53, and *χ*^2^=1, *P*=.43, respectively), ethnicity (Multimedia Appendix 3; *χ*^2^=0.3, *P*=.75, and *χ*^2^=0.03, *P*>.99, respectively), and age (Multimedia Appendix 3; *χ*^2^=3.4, *P*=.64, and *χ*^2^=1.9, *P*=.61, respectively) in both respondent groups. With respect to ethnicity, all ethnicities other than “Welsh/English/Scottish/Northern Irish/British” were defined as minority ethnicities.

### Focused Questions

**Figure 1:**
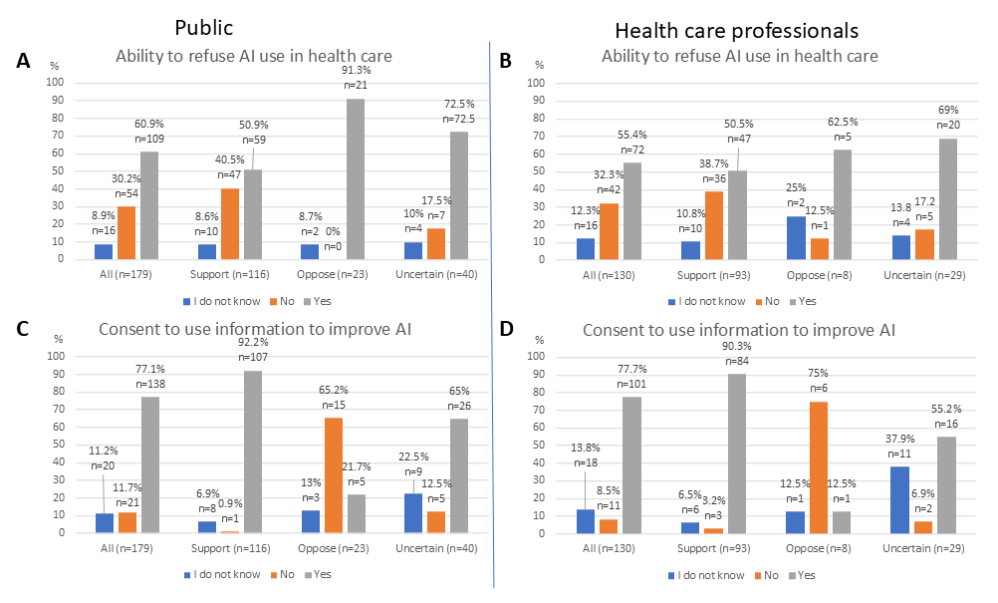
Responses to questions 8 (A), 10 (C), 30 (B), and 32 (D) of the questionnaire presented by the general attitude toward health care AI. AI: artificial intelligence.

Across all groups, most responders stated that patients (members of the public group) and clinicians (health care professional group) should be able to refuse to have AI used in their care or practice. This choice was more pronounced in those with a negative or uncertain self-declared attitude toward health care AI (Figure 1A,B), but a difference between those with a positive and negative attitude was only statistically significant among members of the public (*χ*^2^=14.7, *P*<.001, with post hoc *P*=.001 for “no” and *P*=.002 for “yes” responses; *χ*^2^=2.8, *P*=.24, in the health care professional group).

The overwhelming majority of respondents with a positive self-declared attitude toward health care AI would, in principle, agree to sharing health care data to improve an AI technology that was already used in their care (Figure 1C,D). While skeptics in both groups overwhelmingly objected to such use of health care data (*χ*^2^=81.8, *P*<.001, for members of the public, and *χ*^2^=48.3, *P*<.001, for health care professionals, post hoc *P*<.001 for “no” and “yes” responses in both groups), most of those with an uncertain attitude also agreed with the use of such data to improve existing AI technologies.

**Figure 2:**
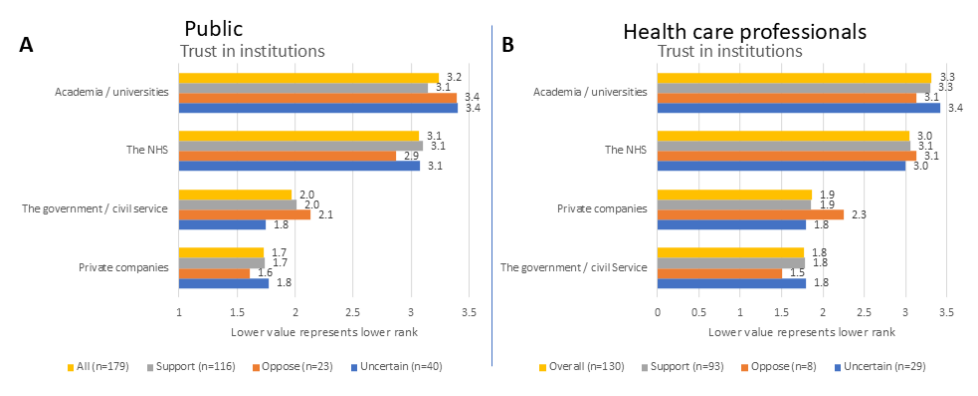
Response to questions 9 (A) and 31 (B) of the questionnaire presented by the general attitude toward health care AI. Items are presented in order of overall rank (highest ranked on top, lowest ranked at the bottom), while bars represent both the overall rank and the responses given by the different AI attitude subgroups. The x-axis denotes the reciprocal of the rank. AI: artificial intelligence; NHS: National Health Service.

Respondents in both groups tended to trust academic and NHS institutions more to develop health care AI technologies when compared to civil service and private organizations (Figure 2C,D). This pattern held true for respondents from all groups of self-declared attitudes toward AI.

**Figure 3:**
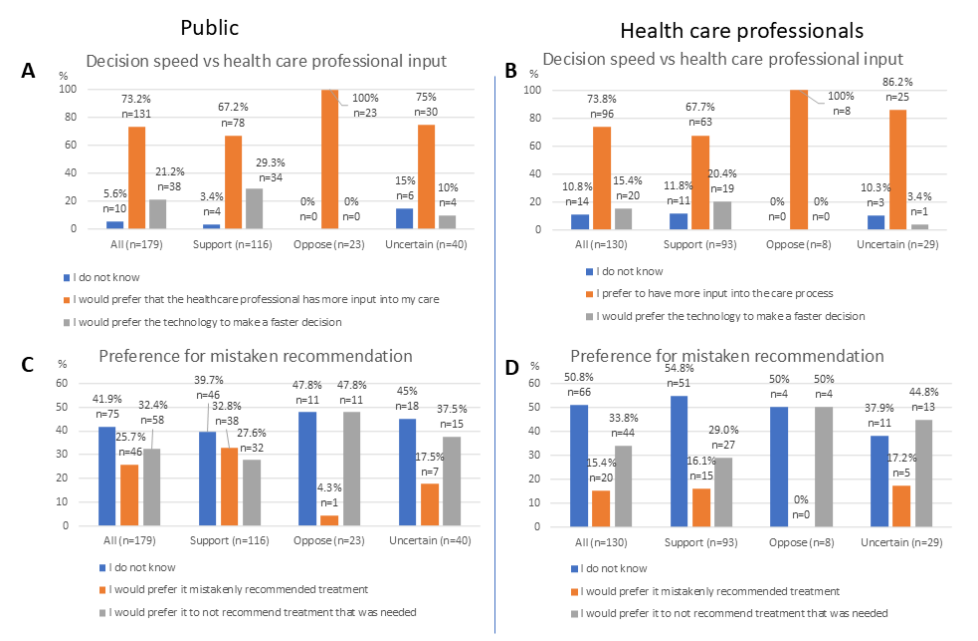
Responses to questions 11 (A), 12 (C), 33 (B), and 34 (D) of the questionnaire presented by the general attitude toward health care AI. AI: artificial intelligence.

Across all groups, responders preferred health care professionals to have more input into the clinical decision-making process over a more automated and speedier process (Figure 3A,B). All respondents from both AI-skeptical groups exclusively selected this answer, but this was only statistically significant in the members of the public group (*χ*^2^=10.4, *P*=.01; *P*>.99 for “I do not know”; *P*=.01 for preference of more health care professional input; and *P*=.02 for preference for faster decision-making); *χ*^2^=3.7, *P*=.15, in the health care professional group.

Overall, there was no clear preference for AI mistakenly recommending treatment or mistakenly not recommending a treatment, as most respondents selected the “I do not know” answer (Figure 3C,D). In both AI-skeptical groups, respondents were evenly split between the “I do not know” answer and preferring the AI to mistakenly not recommend treatment, with only a minority preferring it to mistakenly recommend the treatment. While not as pronounced, a similar pattern was present among the uncertain groups. There was no statistically significant difference between those with a self-declared positive and negative attitude toward AI in the health care professional group (*χ*^2^=2.4, *P*=.29), but there was among the members of the public (*χ*^2^_2_=8.4, *P*=.02), with no significant difference among those who selected “I do not know” (*P*>.99) and those who preferred treatment to be mistakenly not recommended (*P*=.33), but significant differences among those preferring a mistaken treatment recommendation (*P*=.03).

**Figure 4:**
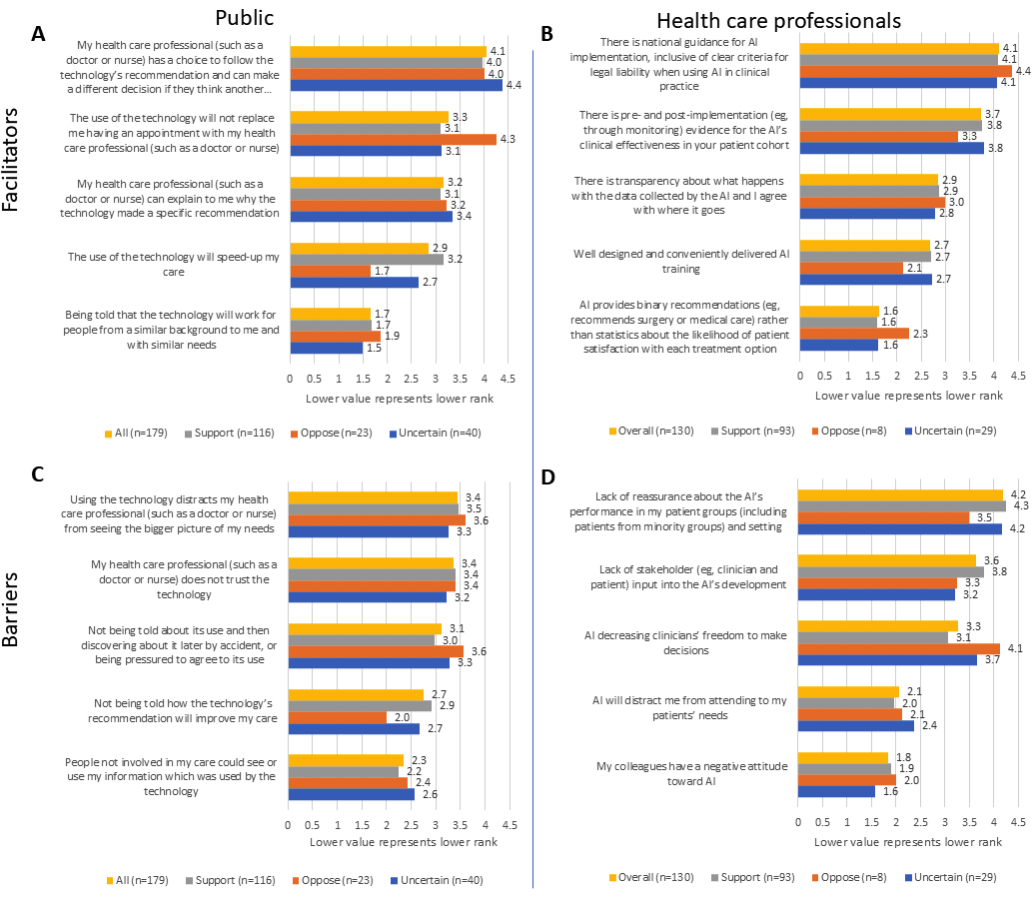
Ranking of various potential facilitators (A and B) and barriers (C and D) to health care AI adoption by members of the public (A and C) and health care professionals (B and D); questions 13, 14, 35, and 36 in the questionnaire. The items are presented in order of overall rank (highest ranked on top, lowest ranked at the bottom), while bars represent both the overall rank and the responses given by the different AI attitude subgroups. The x-axis denotes the reciprocal of the rank. AI: artificial intelligence.

When asked to rank 5 potential facilitators of health care AI, members of the public ranked as the highest option the statement “My healthcare professional (such as a doctor or nurse) has a choice to follow the technology’s recommendation and can make a different decision if they think another option is better” (Figure 4A). The exception was the group of AI skeptics, who, while still highly valuing that answer, scored slightly higher for the answer stating that the use of AI would not replace them having an appointment with a health care professional; this was the overall second-highest-ranked answer among the members of the public. The third highest ranked facilitator was that the health care professional could explain why an AI reached a specific decision. Overall, speeding up care and knowing that the technology worked for people of a similar background were the lowest-scoring facilitators for the members of the public, particularly for the AI skeptics, although AI supporters gave similar preference to the 3 midscoring answers. Among health care professionals (Figure 4B), the highest-ranked facilitator was the presence of national guidance for AI implementation, including on legal liability, and was followed by pre- and postimplementation evidence for the AI’s effectiveness. This was followed by transparency about the destination of the data collected by the AI and clinician agreement as to this destination, followed by conveniently delivered training, and the AI providing binary recommendations. Among health care professionals, there was little variability in the responses given by the 3 AI attitude groups.

Among members of the public, the highest-ranked barrier to AI adoption was that AI might distract the health care professional from the bigger picture of the patients’ needs (Figure 4C). This was followed by a lack of trust in the health care professional in the AI technology. The third highest ranked barrier was if the technology was used without disclosing it to the patient or if the patient felt pressured to have it used in their care. This barrier was somewhat higher ranked by AI skeptics when compared to AI supporters. The last 2 barriers were not being told about the benefits of the use of AI and whether people outside of the patient’s care team would be able to see the information used by the AI. For health care professionals, the highest-ranked barrier was the lack of reassurance about the AI’s performance in their patient group, except for AI skeptics, who ranked as the highest barrier in clinicians’ decision-making freedom, which was overall the third-highest-scoring barrier (Figure 4D). The second-highest overall scoring barrier was the lack of stakeholder input into the AI’s development. This was followed by health care professionals being distracted from their patients’ needs and their colleagues having a negative attitude toward AI.

### Ranking of Ethics Principles

**Figure 5:**
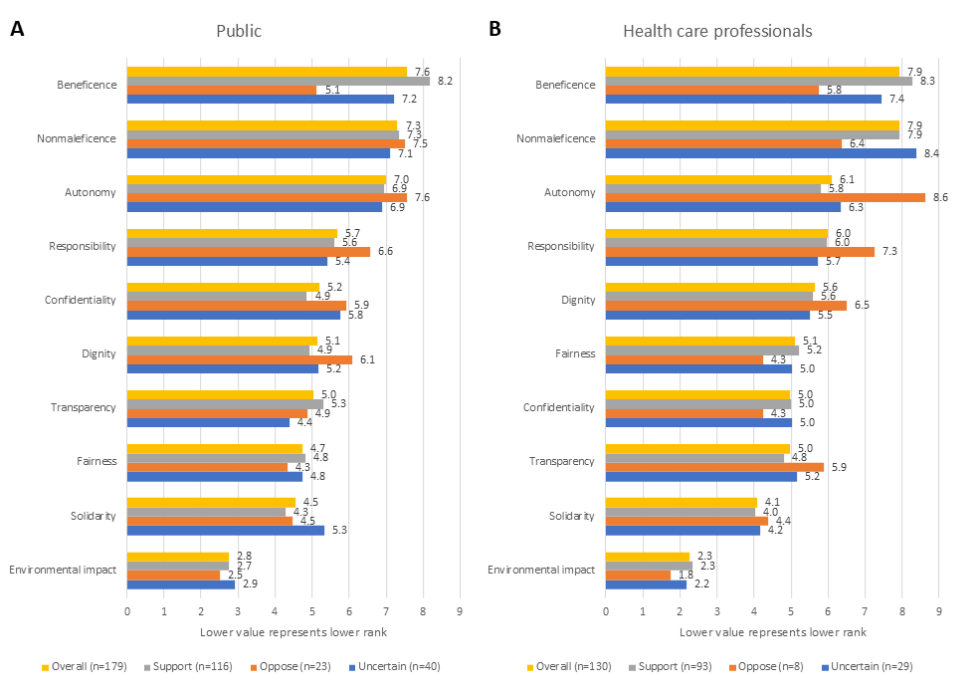
Ranking of health care AI ethics principles (questions 15 and 37 in the questionnaire) by members of the public (A) and health care professionals (B). The principles are presented in order of overall rank for each eligibility criterion group (highest ranked on top, lowest ranked at the bottom), while bars represent both the overall rank and the responses given by the different AI attitude subgroups. The x-axis denotes the reciprocal of the rank. AI: artificial intelligence.

There was a large congruence between members of the public and health care professionals in the ranking of ethics principles relating to health care AI, and the highest-ranked principles and two lowest-ranked principles were the same in both groups (Figure 5A,B). These principles were described slightly differently to the two eligible groups (Multimedia Appendix 3). Moreover, similar trends were seen in the AI skeptic groups in both eligibility criteria groups. AI skeptics ranked autonomy as the most important principle, which overall was the third-highest-ranked principle, while beneficence, which was overall the highest-ranked principle, was ranked only as sixth by AI skeptics. Similarly, dignity and responsibility were consistently ranked higher by AI skeptics than by the overall population. Environmental impact was the lowest-ranked principle among all respondent groups. Solidarity was the overall second-lowest-scoring principle in both the members of the public and the health care professional respondent groups. Nevertheless, for members of the public who are AI skeptics, fairness was the second lowest scoring principle, while for those who were uncertain, fairness, transparency, and dignity scored lower than solidarity. In the health care professional group, AI skeptics scored confidentiality and fairness lower than solidarity, but solidarity was the second lowest scoring principle among those with an uncertain attitude.

### Free-Text Responses

Respondents were asked to state their top barrier and facilitator to the adoption of AI in health care and then two other important barriers and facilitators. As these were free-text fields, respondents stated (including blank and invalid answers) a total of 1054 barriers and 1017 facilitator items, while 927 items were originally anticipated for each. The final list of themes and subthemes used to categorize these responses can be found in Multimedia Appendix 4.

**Figure 6:**
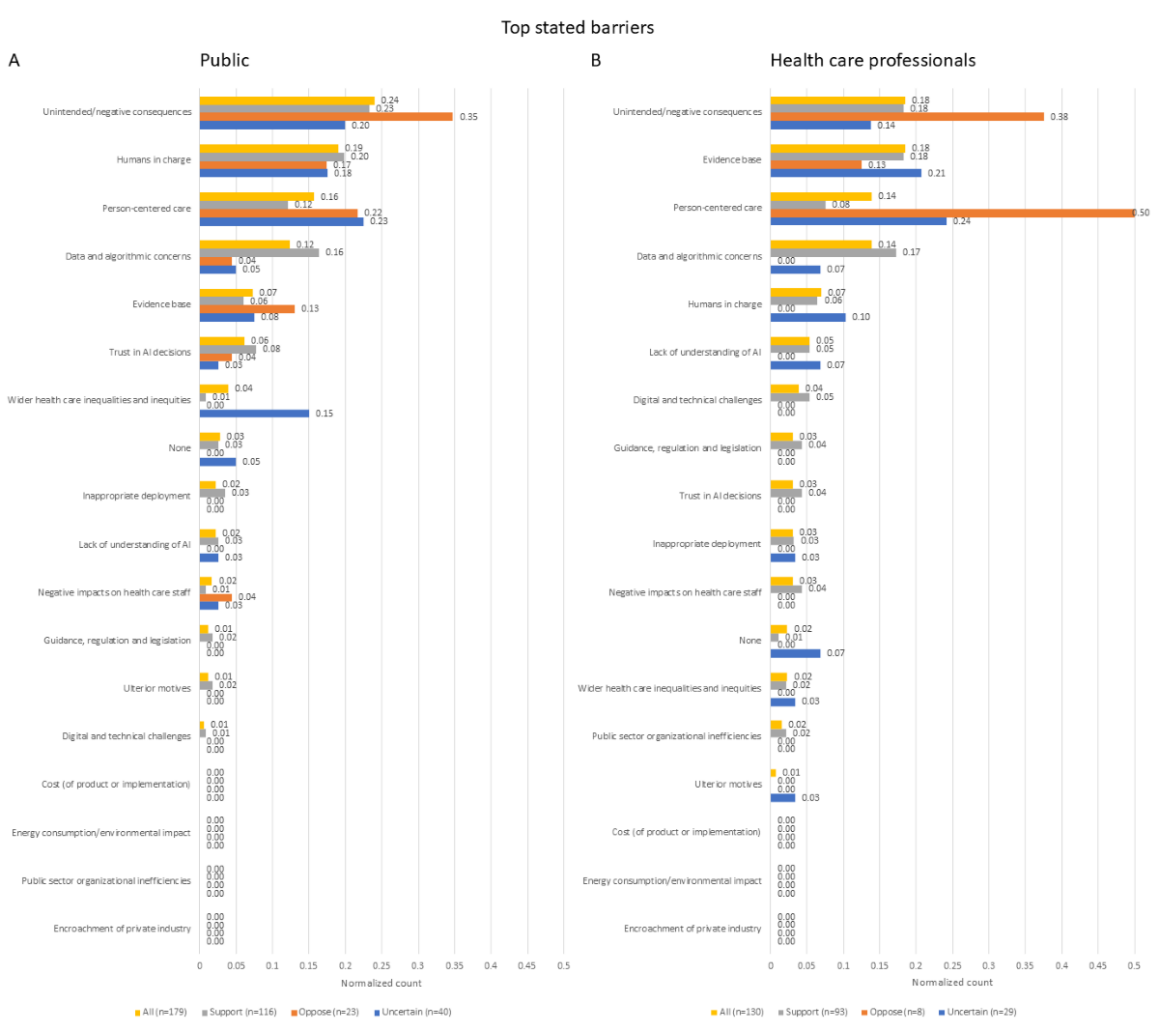
Count of all the most important barrier themes to AI adoption stated by participants and normalized to the number of items stated in each group. n indicates the number of counted items, which in this case is equal to the number of respondents in each group. AI: artificial intelligence.

When looking at the top barrier to adoption (Figure 6), both members of the public and health care professionals have placed the potential for unintended or negative consequences as their most important concern. This theme was particularly popular among AI skeptics in both groups, though health care professional skeptics were even more concerned about the erosion of human-centered care, which was the overall third-ranked barrier. None of the respondents mentioned cost, environmental concerns, or encroachment of private industry as their main concern. There were no statistically significant differences between skeptics and those with a positive attitude toward AI (*χ*^2^=10.7, *P*=.60, for members of the public; *χ*^2^=18.2, *P*=.18, for health care professionals).

When all responses to the 3 barrier questions were accounted for (Multimedia Appendix 3), the top 3 themes among both respondent groups were erosion of person-centered care, potential for unintended or negative consequences, and data and algorithmic concerns, although the last of these was most important for health care professionals. Erosion of person-centered care was the most frequently stated barrier among skeptics. None of the skeptics in either group provided an invalid or blank answer to any of the barrier questions. Among members of the public, there were no statistically significant differences between skeptics and those with a positive attitude toward AI (*χ*^2^=22.8, *P*=.16), but among health care professionals there was a significant difference between these groups (*χ*^2^=32.7, *P*=.01), with post hoc testing showing that issues relating to person-centered care were much more important to skeptics (*P*<.001).

**Figure 7:**
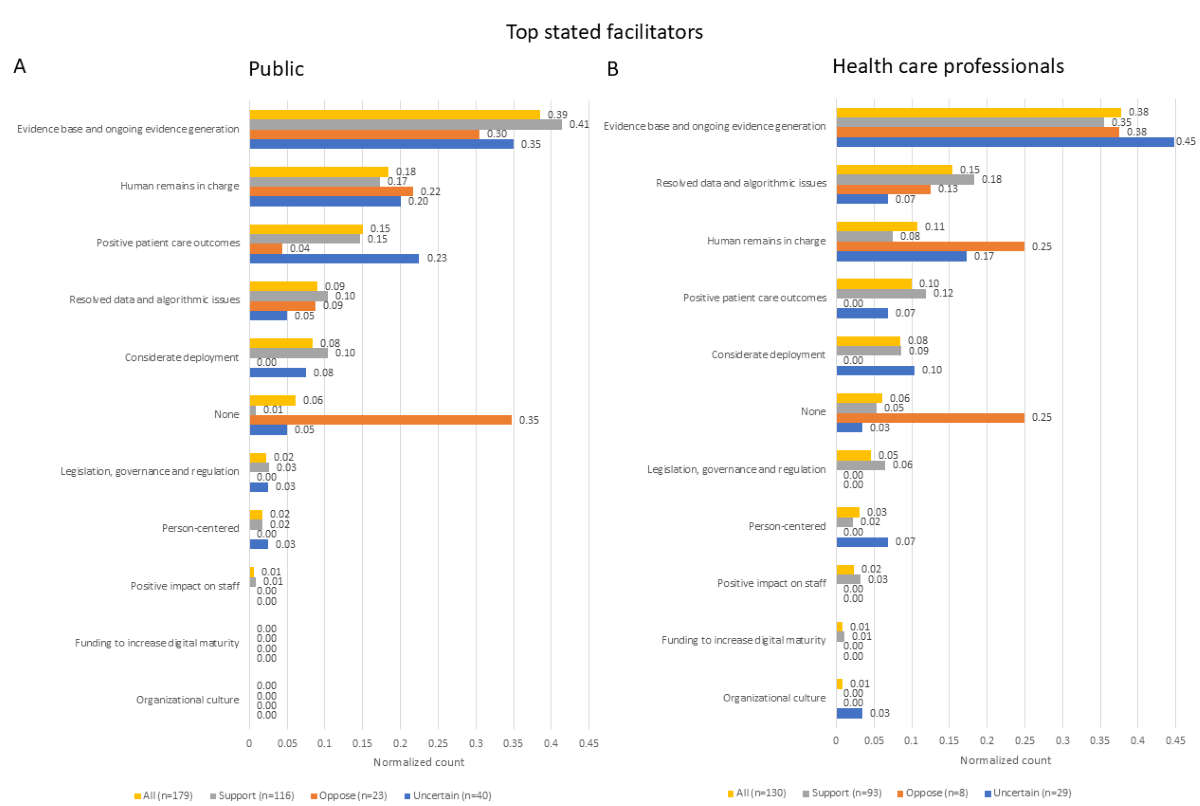
Count of all the most important facilitator themes to AI adoption stated by participants and normalized to the number of items stated in each group. n indicates the number of counted items, which in this case is equal to the number of respondents in each group. AI: artificial intelligence.

The top facilitator for both respondent groups (Figure 7) was the presence of an evidence base and of ongoing evidence generation with respect to health care AI. The overall order of frequency of mentions of each theme was similar for both groups, with the exception that health care professionals placed more importance on resolving data and algorithmic issues, while members of the public placed more importance on humans remaining in charge and ensuring positive patient outcomes. There were no statistically significant differences between AI attitude groups among health care professionals (*χ*^2^=9.4, *P*=.34), but the overall distribution of answers was significantly different among members of the public (*χ*^2^=40.1, *P*<.001), with post hoc analysis showing (*P*<.001) the larger proportion of the “none” group among skeptics.

When all stated facilitators were considered (Multimedia Appendix 3), both respondent groups had the presence of an evidence base and of ongoing evidence generation with respect to health care AI as the most frequently mentioned theme, and humans remaining in charge as the second most frequently mentioned theme. Among members of the public, resolved data and algorithmic issues were the third most mentioned theme, while positive patient outcomes were the fourth most important theme; this was reversed for health care professionals. Noteworthy, for AI skeptics, the most frequent theme was “none,” which consisted of blank or invalid answers, but this was only statistically significant for members of the public (*χ*^2^=66.9, *P*<.001; post hoc *P*<.001). There was a statistically significant difference in answer distribution among health care professionals (*χ*^2^=18.8, *P*=.04), but post hoc analysis did not identify any specific factors as significant.

## Discussion

### Literature Search

The scope of the literature search (Multimedia Appendix 1) was wider than the intended questionnaire scope, as the literature search covered all health care AI, while the questionnaire focused on ML technologies used in treatment and therapy. Making our literature review scope too narrow might have resulted in a low number of identified studies and only informed us as to what was already done in this specific field, while having a broader scope allowed us to consider questions that might not have yet been explored in this specific area, but that were explored in adjacent fields. Moreover, reviewing a wide range of literature allowed us to familiarize ourselves with a broad range of approaches used in similar studies and so inform our survey design.

The literature review highlighted a relative lack of studies focusing on the patient or member of the public perspective on clinical AI adoption (Multimedia Appendix 1). As such, we considered it vital to attend to this perspective and work closely with our PPI representatives to focus the survey on the most pertinent questions to this population.

### Questionnaire Development

When developing the questionnaire, we sought extensive input from our PPI and stakeholder groups, rather than focusing on using a specific technology adoption framework. Our PPI (who were residents of Wales) and stakeholder groups allowed us to identify which questions were most important to ask and allowed us to keep the questionnaire concise to ensure that the survey results would be informative, but the survey itself would not be burdensome. Additional feedback from colleagues and lay volunteers was used to ensure that the questionnaire was understandable to the intended audience. Consequently, while our questionnaire was not validated, it received a similar level of input from experts to a similar study described previously [24]. Moreover, our extensive use of public involvement aligned our approach with recent developments in research methodology and good practice [7,8]. Finally, it was not always clear how to represent certain response options. For example, the primary area of work options presented in Multimedia Appendix 3 were selected based on clinician input, but there are other reasonable alternatives that could have been used.

Nevertheless, after deploying the questionnaire, we received feedback that allowing only single answers to the eligibility criteria question, primary specialisms question, and geographical areas of work question was problematic for those who met both criteria or worked across several specialisms or in more than one health board’s territory. Yet, this should not have a negative impact on the interpretation of the results and since the demographic data is mainly used to ascertain the representativeness of our respondent population. Additionally, because the eligibility question was used to direct respondents to the correct questionnaire stream, a specific set of questions would need to be developed for respondents who met both eligibility criteria, which would likely be most health care professional respondents.

We also received feedback that the specific barriers and facilitators mentioned throughout the survey were not always the most important ones for specific respondents (eg, questions 35 and 36 in Multimedia Appendix 2). While we acknowledge this limitation, the survey was built with stakeholder input, and the free-text questions were specifically included to capture any themes we might have missed in the rest of the questionnaire.

### Respondent Demographics

Considering Wales’ relatively small population of approximately 3.2 million, 309 responses represent a relatively high response rate compared to other similar studies conducted in other countries [24,25]. The main deviations of the responder population from the reference population were the underrepresentation of women and overrepresentation of men among respondents in the health care professional group (despite most respondents being women), and overrepresentation of members of the public respondents from the Cardiff and Vale University Health Board area. We are not sure why women were underrepresented in the health care professional group, but the overrepresentation of respondents from the Cardiff and Vale University Health Board area is likely related to our research team being based in Cardiff, making it easier to advertise the study to those living and working in this area.

To assess the impact of this, we undertook an additional analysis comparing the responses of women and men in the health care professional group to questions 30, 32, 33, and 34 (Multimedia Appendix 2), with the only statistically significant difference (*χ*^2^_2_=9, *P*=.01; post hoc *P*=.02) being the increased proportion of “I do not know” responses among women to question 30 (asking about the right of clinicians to object to using health care AI technologies in their clinical practice). We also explored the profile of members of the public living in the Cardiff and Vale University Health Board area, finding that most responses from ethnic minority persons were from this location (22 out of 30 ethnic minority respondents). Consequently, we did not apply weights to the responses as the impact of this would be unlikely to be meaningful for the health care professional group, and we did not wish to disenfranchise ethnic minority responders from the members of the public group, as ethnic minorities are usually regarded as underrepresented in research [43]. Nevertheless, while we acknowledge that a higher response rate from those living in rural areas would have been preferable, we did manage to get responses from those living in all Welsh counties.

Finally, it is important to highlight that convenience sampling is associated with an array of limitations. Purposive sampling, together with a compensation for participation, could have potentially overcome these limitations, but resources for this were not available. Additional discussion regarding demographic data can be found in Multimedia Appendix 3.

### Support, Opposition, and Ambivalence to Health Care AI

Most respondents from both the members of the public and health care professionals’ groups self-reported themselves to be in general support of health care AI (Table 1), and 22% of respondents in both groups described themselves as “uncertain.” Among health care professionals, 6% declared themselves as opposed, corresponding to 8 responses, while for members of the public, it was 13% corresponding to 23 individuals. The small size of the skeptic groups also affected the power and certainty around the conclusions derived from statistical hypothesis testing. Nevertheless, chi-square tests indicate that there were no significant differences between respondent demographics from all 3 AI attitude categories within the 2 eligibility groups (Multimedia Appendix 3).

The low response rate from those generally opposed to health care AI was discussed with the PPI and stakeholder group members, with several possible reasons proposed. If skeptics were generally averse to digital technologies, they might not have found out about the survey. Furthermore, skeptics might have chosen not to answer these questions because they could have felt that they did not have enough knowledge to even attempt it. For those skeptics fearful of AI, for example, taking over their jobs or because of the negative news stories surrounding AI in relation to deepfakes, responding to such a survey might be seen as potentially validating any conclusions from the survey, even if their personal responses would not agree with the majority. An analogy was drawn with organizations not taking part in consultations, as if they do take part, they are simply listed as having taken part in it without noting their disagreement with the consultation’s consultation. AI might also be seen as being pushed on the population, which might motivate some to exhibit contrarian attitudes toward it and not participate in anything relating to AI. Such an attitude of suspicion was even evident among some skeptical respondents, with one responding with “Nothing really. I dislike dystopian healthcare!” when asked to state a facilitator. Alternatively, the population of AI skeptics might be small, and our small proportion of skeptics is reflective of the wider population. Whatever the reason for this low proportion of skeptical respondents, this limits the amount of insight this survey can generate in relation to that population, and certainly with which these results can be used to derive practices that make AI more acceptable to that population.

The answers to the other questionnaire items corroborated the self-stated attitude toward health care AI. For example, skeptic respondents were predominantly in favor of the ability to object to the use of AI in health care (Figure 1A,B), with no members of the public opposing the right to such an objection, and skeptics were opposed to the use of their data to improve an AI, which was contrary to the trend displayed by those supporting AI or being ambivalent to it (Figure 1C,D). Similarly, AI skeptics often did not list any facilitators to AI adoption (Multimedia Appendix 3), potentially demonstrating that their opinion is set and unlikely to change. The distinct response pattern of skeptics, even when compared to the uncertain respondents, indicated that it would not have been insightful to combine these two groups for the purposes of statistical analysis; for example, Figure 5 shows that the response patterns of the ambivalent groups are more like those of supporters than of skeptics.

### Barriers and Facilitators

A general preference for the ability of patients and clinicians to refuse the use of AI in health care (Figure 1A,B) and for more health care professional input during the care process (Figure 3A,B) is consistent with a high ranking of autonomy among the 10 ethical principles (Figure 5A,B). This is also reflected in the ranking of specific barriers and facilitators (Figure 4A,C,D), and is particularly prominent among those with a negative attitude toward health care AI. In the free-text answers, prioritizing themes of humans remaining in charge and concerns about person-centered care (Figures 6 and 7 and Multimedia Appendix 3) also reflected this, although the latter also relates to the ethical principle of dignity (Figure 5). As such, to facilitate adoption automation of specific tasks, rather than the overall care process, and ensuring that a human stays in the loop should be ensured. While facilitating consent to AI-augmented interventions seems desirable, this might not be feasible if AI-augmented practice becomes the standard of care [44].

The higher trust in the NHS and academic institutions to develop health care AI, when compared to civil service and private companies (Figure 2), was to a certain degree reflected in the free-text answers, though with more nuance. Themes of public sector organizational inefficiencies, the encroachment of private industry, ulterior motives associated with AI deployment, and a specific subtheme regarding the trustworthiness of developers and that a broad stakeholder group should be involved in the development of these technologies (Multimedia Appendix 3) have highlighted the complexities of this issue. As one respondent stated when asked to list a facilitator:

If the AI was developed by a private non-profit, where the development of this would be open source. This would free the company from the bureaucracy, corruption and sheer incompetency that the government and NHS management are best at, (let's leave that to them). Secondly, a non-profit company would be focused on the work and not motivated by profit, being open source would allow for public scrutiny.

These results not only support cooperation between the NHS and academic institutions developing such technologies, but also the role of not-for-profit organizations, which might be able to overcome some hurdles faced by the public sector organizations, avoid concerns regarding profit prioritization, and foster trust by using open-access standards.

Overall, most people were happy to have their information shared to improve the way an AI technology works, except for skeptics who overall opposed the use of their data in such a way (Figure 1C,D). This might relate to the principle of confidentiality (Figure 5), which was more important for skeptics who were members of the public. While data and algorithmic concerns were the most often mentioned overall barrier (Multimedia Appendix 3), some responses indicated that people wanted their data to be used to help others, which was reflected in the subtheme of AI updating and improvement (Multimedia Appendix 3). There is a potential interplay here with concerns about how private companies might use patient data and whether it will cause private enterprise to profit, rather than benefit the patients and the NHS. It is uncertain if AI skeptics would be more willing to share their data for the purposes of AI improvement if the AI technology was owned by the NHS or academia.

There was no clear preference for whether AI should mistakenly recommend or not recommend treatment (Figure 3C,D), but the themes of potential harms and benefits were prominent in responses to other questions. Beneficence and nonmaleficence were the two overall highest-ranked ethical principles (Figure 5A,B), and unintended or negative consequences of AI were the most often mentioned top barrier (Figure 6). While positive patient outcomes themselves were often stated as an important facilitator, the related theme of evidence basis and ongoing evidence generation was the top-stated facilitator (Figure 7 and Multimedia Appendix 3). Yet, the picture given by Figure 4 is somewhat more complex. The specific benefit of faster care was the second lowest rated facilitator for members of the public (Figure 4A), while the presence of national guidance was rated higher in that question as a facilitator than the presence of evidence for the effectiveness of the technology in the clinician’s patient cohort (Figure 4B). Similarly, patients ranked not being told about how a technology will improve their care as the second least important barrier (Figure 4C), although health care professionals ranked the lack of evidence of the AI’s performance in their patient group as the highest scoring barrier (Figure 4D). This highlights that knowledge about the potential benefit of the technology is important, but the exact importance is uncertain.

It is worth noting that many of the themes identified were multifaceted. For example, accountability (Multimedia Appendix 3), corresponding to the ethical principle of responsibility (Figure 5), encompassed concerns of members of the public about who will be responsible for harms resulting from AI-enhanced care, but also of health care professionals wanting to know the extent of their liability arising from mistakes caused by an AI. Moreover, there were concerns that clinicians might start blaming AI for their own mistakes and that the government might blame the AI for their own failures. Furthermore, while patients and clinicians emphasized the need for freedom to reject AI recommendations, there were also worries that clinicians would reject AI recommendations without a good reason. This could be linked with responses that required an AI to be 100% accurate before being allowed to be used clinically, and the recognition that an AI technology might be able to spot things that a clinician might otherwise not notice. Finally, while some health care professionals expressed worries that patients might leverage AI against them, members of the public were concerned that AI might be used to “gaslight” patients with difficult presentations, but also expressed hope that AI might reduce the possibility of clinician judgment error. This shows that both patients and clinicians are often concerned about similar problems, and that while there are worries that the introduction of AI into health care might depersonalize care, there is also an appreciation that AI might alleviate some problems and should not be outright dismissed.

### Theoretical Contributions

When comparing this survey’s findings to the TAM and UTAUT principles [4], the key barriers and facilitators in our study were those relating to the perceived usefulness or performance of the technology (Table 2). Those relating to subjective norms were also prominent, mostly manifesting in the need to maintain human interaction during the health care encounter. The other TAM and UTAUT principles, while not completely absent, were not as strongly present among respondents' answers. This supports the case for implementing digital technologies only if they can improve the health care process and outcomes, rather than only because an algorithm can perform a task with high accuracy or because a technology can be implemented for implementation’s sake. Furthermore, the results show that members of the public and clinicians do not want the human element of care to be eliminated. While some might see the implementation of digital health care technologies as an opportunity to decrease the amount of health care staff input into patient care, which is often a large part of health care costs, this survey’s results caution against such an approach. While digital health care technologies will hopefully speed up patient care, the survey results suggest that improving the quality of care is more likely to be a more acceptable use of the time savings, for both members of the public and staff (Figures 6 and 7; Multimedia Appendix 3).

**Table 2:** Comparison between TAM^a^ and UTAUT^b^ principles and this study’s findings.

TAM or UTAUT principle	Examples from this study	Comments
Perceived ease of use or individual effort expectancy	Negative impact on health care staff, lack of understanding of artificial intelligence, digital and technical challenges, considerate deployment	Several themes related to this technology adoption principle, but none were prominent among respondents Does not relate directly to any ethics principle
Perceived usefulness or performance expectancy	Unintended or negative consequences Evidence base and ongoing evidence generation Beneficence and nonmaleficence	Top barrier among both respondent groups Top facilitator among both respondent groups Top 2 ethics principles among both respondent groups
Subjective norms or habits	Humans (remain) in charge and person-centered care Autonomy, responsibility, and dignity	Both in the top 3 overall stated barriers and facilitators among both respondent groups, and at least one of the two in the top stated barriers and facilitators The first 2 ethics principles were in the top 4 ranked principles in both groups, while dignity was fifth (health care staff) and sixth (public)
Social influence	Evidence base and ongoing evidence generation Organizational culture	Several themes related to this technology adoption principle, but none were prominent among respondents Does not relate directly to any ethics principle
Facilitating conditions	Legislation, governance and regulation, funding to increase digital maturity, inappropriate deployment, considerate deployment	Several themes related to this technology adoption principle, but none were prominent among respondents Does not relate directly to any ethics principle

^a^TAM: Technology Acceptance Model.

^b^UTAUT: Unified Theory of Acceptance and Use of Technology.

While Jobin et al [29] identified transparency and fairness as the two most frequently mentioned principles among AI guidelines, our results suggest that with regard to health care AI, these are not the most important principles among end users of such technologies. Beneficence and nonmaleficence were the two highest-ranked principles among both our respondent groups (Figure 5), followed by autonomy and responsibility; nonmaleficence and responsibility had joint third place in the ranking by Jobin et al [29]. These differences between our respondent preferences and the findings by Jobin et al might potentially relate to the ease of expressing transparency requirements in the form of structured guidelines and legislation, while beneficence and nonmaleficence are harder to implement due to the dependence of the realization of these principles on how a technology is implemented in an organization, how end users apply it, and how (in the case of AI) the training population matches the patient population. Nevertheless, our data suggest that beneficence and nonmaleficence should be at the forefront of AI implementation guidelines.

Finally, it is worth briefly relating our findings to other similar international studies. Our findings broadly correlate with Isbanner et al [24], who reported in their Australian study that respondents were happy for health care to be augmented through the use of AI but did not want humans to be replaced in the care process. Interestingly, among their respondents, 13.4% stated that they “somewhat” or “strongly” oppose the development of AI, which is similar to the 13% recorded in our responses from members of the public (Table 1), suggesting that our small proportion of respondents opposed to AI might match trends in other culturally similar countries to Wales. Similar to our findings about the importance of the perceived usefulness or performance of the technology, a recent report from Germany by Kühne et al [25] found that technology reliability was the main factor affecting technology acceptance. While respondents in the study by Kühne et al [25] placed less emphasis on autonomy, similar to our study and that by Isbanner et al [24], respondents preferred AI systems that work collaboratively with humans over those that replace human input. As such, our study’s results are largely in congruence with international findings.

### Limitations

While there are some other limitations of this study, such as not having validated the questionnaire in a smaller population before deployment, not assessing the relationship of the questionnaire items using Cronbach α, or not using snowball sampling during the literature review, here we focus on the 3 main limitations of this study.

The main limitations of this study are the use of convenience sampling, a high proportion of respondents from the Cardiff and Vale area, and a low number of respondents who are generally opposed to the use of AI in health care. Because of the anonymous nature of the survey, we were also unable to check for duplicate responses. Nevertheless, the study achieved a good representativeness of the population of Wales. To minimize the risk of the voice of AI skeptics being lost, we presented the data split by overall attitude. Their responses are particularly important as specific approaches might need to be implemented to encourage them to use ML-based approaches in their care.

While the survey intended to focus on the application of ML to facilitate prudent health care in Wales, as exemplified by the case study presented in the questionnaire (see Multimedia Appendix 2), the respondents often addressed broader issues with respect to the application of AI technologies in health care. This has been particularly evident from free-text responses mentioning large language models or interacting with chatbots. This is unsurprising given the prominence of these types of technologies, for example, ChatGPT, in the media (Multimedia Appendix 4). Hence, respondents might have used this survey as an opportunity to express their concerns regarding the broader application of health care AI. Consequently, the findings from this survey are likely to be applicable to a broader range of health care AI technology implementation scenarios than those focusing solely on prudent health care. Yet, this also means that the results less reliably represent the views of respondents on this specific topic.

Lastly, the survey did not explicitly target other stakeholder groups, which are vital for the adoption of new health care technologies, such as health care leaders, government managers, as well as information technology and governance specialists. These people could nevertheless fill out the questionnaire if they met the inclusion criteria. Nevertheless, diffusion of innovation theory suggests that since members of the public and clinicians are the final adopters of health care technology, it is they who primarily need to be convinced of a technology’s appropriateness [1].

### Future Work

This survey did not include any questions that tried to address why any specific barriers and facilitators were deemed important. The next step of this project is to conduct a range of focus groups to address this question and to engage members of underrepresented groups. Information gathered from focus groups, together with this survey’s data, will allow for the formation of policy recommendations.

Research targeting health care leaders, policymakers, and informaticians would help describe the challenges of AI adoption from an organizational perspective [2], although individual NHS Wales organizations make their own adoption decisions for specific digital technologies.

### Conclusions

Based on the responses from both members of the public living in Wales and health care professionals participating in treatment or therapy decision-making and working in Wales, there is a strong preference for ensuring that AI technologies are assessed for their effectiveness and that these technologies do not replace human input into the care process. Moreover, there is a clear hesitancy toward the introduction of commercial technologies and a preference for developing these with strong clinical and academic input. Consequently, developing technologies locally by health boards or trusts, or via DHCW, together with robust internal evaluation, might present the way forward. While such an approach might not be feasible for some digital applications, it might be particularly suitable for ML applications within the context of prudent health care, where algorithms are likely to require training on local population data. Moreover, for such solutions to be acceptable, it is important that the results are reviewed by a human clinician and that patient-clinician contact is not decreased due to the introduction of these technologies, even if this reduces the cost-savings that could result from the introduction of such technologies.

## Appendix

Multimedia Appendix 1 Literature review.

Multimedia Appendix 2 Survey questionnaire.

Multimedia Appendix 3 Additional tables and figures.

Multimedia Appendix 4 Google search statistics as of June 21, 2025.

Multimedia Appendix 5 CHERRIES checklist.

## Abbreviations

AI: artificial intelligence
CHERRIES: Checklist for Reporting Results of Internet E-Surveys
DHCW: Digital Health and Care Wales
ML: machine learning
NHS: National Health Service
NICE: National Institute for Health and Care Excellence
ONS: Office for National Statistics
PPI: patient and public involvement
PROM: patient-reported outcome measures
TAM: Technology Acceptance Model
UTAUT: Unified Theory of Acceptance and Use of Technology

## Data Availability

The datasets generated or analyzed during this study are not publicly available due to the study’s information governance arrangements. The data can be requested from the corresponding author, and the request will be assessed for compliance with the study’s information governance arrangements.
