# Supplementary material for: Barriers and Facilitators to Health Care AI Adoption Among Those Living in Wales and Working in Health Care in Wales: Online Survey
Source: J Med Internet Res. 2025 Dec 5;27:e81543. doi: 10.2196/81543 (PMC12717503; doi:10.2196/81543)
Supplement: Multimedia Appendix 1 [file jmir_v27i1e81543_app1.docx]

# Literature review

**Review scope**

|  | **Inclusion** | **Exclusion** |
| --- | --- | --- |
| **Population** | Implementation of AI in any area of healthcare | Healthcare education |
| **Phenomenon of interest** | Factors which support or hinder the adoption of healthcare AI across a range of key stakeholders, such as patients/public/caregivers, clinical staff, management staff, healthcare institutions’ IT staff, and other key healthcare decision-makers (e.g. government).  Guidelines for the adoption of AI in healthcare |  |
| **Outcome** | - Barriers to the implementation or acceptability of AI in healthcare - Facilitators to the implementation or acceptability of AI in healthcare | - Technical specifications of acceptability, e.g. meeting a specific informatics standard for interoperability (rather than interoperability in general) or a specific cut-off value for AI accuracy |
| **Study design** | Any study design (including quantitative, qualitative, mixed-methods) which contains empirical data  Reviews utilising a high-quality search strategy  Thesis | Books  Narrative Reviews  Opinion pieces  Case studies (unless they include empirical data) |
| **Year** | 2000 onwards | Studies published before 2000 |
| **Language** | English language | Not English language |

**Search strategy**

Ovid MEDLINE(R) ALL <1946 to October 10, 2023>

| **#** | **Query** | **Results from 11 Oct 2023** |
| --- | --- | --- |
| 1 | exp Artificial Intelligence/ | 180,583 |
| 2 | exp Random Forest/ | 354 |
| 3 | exp Neural Networks, Computer/ | 61,328 |
| 4 | (Artificial* Intelligen* or AI or machine learning or deep learning or neural network* or convolutional network*).tw. | 243,581 |
| 5 | 1 or 2 or 3 or 4 | 326,553 |
| 6 | (imag* or patholog* or radiolog*).tw. | 2,569,252 |
| 7 | (Healthcare or Health Care).tw. | 735,683 |
| 8 | (Medic* or clinic*).tw. | 6,955,125 |
| 9 | 6 or 7 or 8 | 8,937,256 |
| 10 | (implement* or introduc* or adopt*).ti. | 164,248 |
| 11 | (barrier* or facilitat* or enable* or deter* or promot* or imped* or hindrance? or hinder* or driv* or restrain* or challeng*).tw. | 8,093,762 |
| 12 | 5 and 9 and 10 and 11 | 615 |
| 13 | exp animals/ not humans.sh. | 5,161,760 |
| 14 | 12 not 13 | 612 |
| 15 | limit 14 to (english language and yr="2000 -Current") | 582 |

Embase <1974 to 2023 October 10>

| **#** | **Query** | **Results from 11 Oct 2023** |
| --- | --- | --- |
| 1 | artificial intelligence/ | 64,017 |
| 2 | artificial intelligence software/ | 424 |
| 3 | random forest/ | 25,084 |
| 4 | convolutional neural network/ | 26,281 |
| 5 | artificial neural network/ | 52,276 |
| 6 | (Artificial* Intelligen* or AI or machine learning or deep learning or neural network* or convolutional network*).tw. | 293,342 |
| 7 | 1 or 2 or 3 or 4 or 5 or 6 | 336,424 |
| 8 | (imag* or patholog* or radiolog*).tw. | 3,546,491 |
| 9 | (Healthcare or Health Care).tw. | 1,018,609 |
| 10 | (Medic* or clinic*).tw. | 9,868,036 |
| 11 | 8 or 9 or 10 | 12,368,822 |
| 12 | (implement* or introduc* or adopt*).ti. | 211,967 |
| 13 | (barrier* or facilitat* or enable* or deter* or promot* or imped* or hindrance? or hinder* or driv* or restrain* or challeng*).tw. | 10,135,934 |
| 14 | 7 and 11 and 12 and 13 | 673 |
| 15 | limit 14 to (english language and yr="2000 -Current") | 653 |

Cochrane Library

Search Name: AI Barriers and Facilitators

Date Run: 11/10/2023 14:33:36

Comment: AI Barriers and Facilitators - excludes trials

ID Search Hits

#1 MeSH descriptor: [Artificial Intelligence] 1 tree(s) exploded 2942

#2 MeSH descriptor: [Random Forest] 1 tree(s) exploded 54

#3 MeSH descriptor: [Neural Networks, Computer] 1 tree(s) exploded 537

#4 (Artificial* Intelligen* or AI or machine learning or deep learning or neural network* or convolutional network*):ti,ab,kw 12115

#5 #1 or #2 or #3 or #4 13458

#6 (imag* or patholog* or radiolog*):ti,ab,kw 198975

#7 (Healthcare or Health Care):ti,ab,kw 147828

#8 (Medic* or clinic*):ti,ab,kw 1159900

#9 #6 or #7 or #8 1251970

#10 (implement* or introduc* or adopt*):ti,ab,kw 171589

#11 (barrier* or facilitat* or enable* or deter* or promot* or imped* or hindrance? or hinder* or driv* or restrain* or challeng*):ti,ab,kw 467498

#12 #5 and #9 and #10 and #11 845

#13 #12 in Cochrane Reviews with Cochrane Library publication date Between Jan 2000 and Nov 2023, in Cochrane Reviews

Scopus

(TITLE(( "artificial* intelligen*" ) OR ( ai ) OR ( "machine learning" ) OR ( "deep learning" ) OR ( "neural network*" ) OR ( "convolutional network* " ) OR ( "random forest* " )) AND ABS(( patholog* ) OR ( radiolog* ) OR ( healthcare ) OR ( "health care" ) OR ( medic* ) OR ( clinic* )) AND TITLE(( implement* ) OR ( introduc* ) OR ( adopt* )) AND ABS(( barrier* ) OR ( facilitat* ) OR ( enable* ) OR ( deter* ) OR ( promot* ) OR ( imped* ) OR ( hindrance* ) OR ( hinder* ) OR ( driv* ) OR ( restrain* ) OR ( challeng* ))) AND PUBYEAR > 1999 AND PUBYEAR < 2025 AND ( LIMIT-TO ( LANGUAGE,"English") )

IEEE Xplore

(( "artificial intelligence" ) OR ( ai ) OR ( "machine learning" ) OR ( "ML" ) OR ( "deep learning" ) OR ( "neural network" ) OR ( "convolutional network" ) OR ( "random forest" ) ) AND (( patholog* ) OR ( radiolog* ) OR ( healthcare ) OR ( "health care" ) OR ( medical ) OR ( clinical )) AND (("Document Title":implement* ) OR ("Document Title":introduc* ) OR ("Document Title":adopt* )) AND (( barriers ) OR ( facilitat* ) OR ( enable* ) OR ( deter* ) OR ( promot* ) OR ( hinder ) OR ( challenges ))

*Note: the search was restricted to results from 2000*

ACM Digital Library

[[Abstract: "artificial intelligence"] OR [Abstract: ai] OR [Abstract: "machine learning"] OR [Abstract: "ml"] OR [Abstract: "deep learning"] OR [Abstract: "neural network"] OR [Abstract: "convolutional network"] OR [Abstract: "random forest"]] AND [[Abstract: patholog*] OR [Abstract: radiolog*] OR [Abstract: healthcare] OR [Abstract: "health care"] OR [Abstract: medic*] OR [Abstract: clinic*]] AND [[Title: implement*] OR [Title: introduc*] OR [Title: adopt*]] AND [[Abstract: barrier*] OR [Abstract: facilitat*] OR [Abstract: enable*] OR [Abstract: deter*] OR [Abstract: promot*] OR [Abstract: imped*] OR [Abstract: hindrance*] OR [Abstract: hinder*] OR [Abstract: driv*] OR [Abstract: restrain*] OR [Abstract: challeng*]] AND [E-Publication Date: (01/01/2000 TO *)]

*Note:* *Searched The ACM Guide to Computing Literature (3,579,556 records)*

**Study selection flow diagram. Adapted from PRISMA.**[1,2]

Reports excluded: (n = 60)

Lack of relevant outcomes (n = 26)

Conference abstracts (n = 12)

Other reviews (n = 9)

Focused on adoption frameworks (n = 4)

Did not focus on clinicians or patients (n = 4)

Protocols (n = 4)

Did not involve AI (n = 1)

Records identified from databases (n = 2044):

Medline All (n = 582)

Embase (n = 653)

Cochrane Library (n = 12)

Scopus (n = 395)

IEEE Xplore (n = 233)

ACM Digital Library (n = 169)

Records removed *before screening*:

Duplicate records removed (n = 730)

Records screened

(n = 1314)

Records excluded

(n = 1179)

Reports sought for retrieval

(n = 135)

Reports not retrieved

(n = 1)

Reports assessed for eligibility

(n = 134)

Studies included in review

(n = 74)

Reports of included studies

(n = 74)

**Identification of studies via databases and registers**

**Identification**

**Screening**

**Included**

**Barriers and facilitators to healthcare staff adoption of clinical AI technologies.**

| **Theme** | **Descriptor** | **References** |
| --- | --- | --- |
| Performance | Performance of the AI algorithm in terms of factors such as accuracy and safety. How useful it is clinically. | [3–54] |
| Data | Encompasses both issues concerning the data upon which the AI technology was developed and how it utilises patient data obtained from clinical care. | [8–10,12,15–17,21,24,28–32,34,35,41,43,46,50–52,55–59] |
| AI Education | Staff education about AI technologies in general, and the specific technologies they use. | [3,4,7–9,12–14,16,17,19,20,22,24,29,32,35,37,42–44,46–49,51–55,57,59–61] |
| Social Influence | Subjective norms and opinions of one's peer group, as well as that of one's patients. | [3,6–8,12,19–21,29,32,40,41,46,48,51,61] |
| Resource Availability | Financial, infrastructure and technical resource availability, as well as the presence of technical staff and costs. | [3,4,8,10,12,14,17,19–21,23,26,27,29,30,32,34,35,37,40,41,47,50,53,55–59,61,62] |
| Workflow Impact | Impact on clinician workload, workflows, and patient care pathways. | [4–6,10–12,14–30,33–38,41–43,45–50,52–54,56,58,60–64] |
| Clinician Input | Early clinician/peer input into the technology’s development and implementation. | [4,10,11,14–19,22,24,26,30,35–37,45,46,48,50,52–55,59,60,65,66] |
| Situational Purpose | Whether the technology is used in mundane or grey area cases, or all circumstances. | [9,16,18,24,25,33,40,41,44–47,50–52,61,63,66] |
| Autonomy of Practice | The effect of the AI technology on clinician job autonomy and satisfaction. | [6,8,14,18,19,25,28,30,31,33,44–48,50–53,58,60,61,63,66,67] |
| Team Work | How it affects collaboration between various teams and within teams, and how this is affected by communication. | [14,19,20,46,49,53,68] |
| Explainability | Understanding why the AI technology gives specific recommendations. | [5,8,10,11,14,16–19,24,30,31,33,34,37,38,41,46,49–52,56,64,69] |
| Action Thresholds | Set-up of the technology to trigger actions at specific thresholds. | [10,14,17,19,20,23,24,27,28,49,66,69] |
| Usability | Customisability of the technology to suit requirements. | [14,26,34,37,41,45,58] |
| Corporate and Governmental Culture | Effect of 'higher-up' decisions on clinicians’' desire to adopt a technology. | [8,12,14,17,19–21,29,32,35,40,43,47,48,51–53,55,59,62] |
| Accountability | Accountability of practitioners for the use of AI technologies and the legal and best-practice frameworks that promote this. | [6,8,11,12,16–21,24,26,28,29,32,35,40,41,50,51,53,55,56,58–60,66,67,69] |
| Human Contact | Effect of AI technologies on the human aspect of clinical care, such as emotional sensitivity and patient contact. | [20,26,28,51,52,58,64,66] |
| Managing Expectations | How an AI technology works compared to what the expectation was for it. | [10,15,19,25,35,37,46,53,57,60] |
| General AI Attitude | Clinicians' general attitudes towards digital technologies. | [6–8,14,17,19,20,22,29,32,34,37,38,46,47,51,53,56,57,64] |
| Implementation Manner | The way how a technology was deployed/imposed on the clinical team. | [17,19,20,24,33,46,47,52] |
| Vendor Difficulty | Challenges arising from the relationship between staff/organisation and different healthcare technology vendors. | [12,19,25,31] |
| Contracting Challenges | Specific challenges associated with setting up contracts regarding the AI technology. | [37] |
| Effects of Service Inequalities | The effect of the AI technology on local and global inequalities. | [40,50] |

**Barriers and facilitators to patient adoption of clinical AI technologies.**

| **Theme** | **Descriptor** | **References** |
| --- | --- | --- |
| Performance | Performance of the AI algorithm in terms of factors such as accuracy and safety. How useful it is for patients. | [27,43,60,70–73] |
| Time | How the use of the AI affects the length of the clinical encounter. How time-demanding is the use of the AI. | [27,43,70,74] |
| Social Influence | Subjective norms and opinions of one's peer group, as well as that of the clinician. | [27,51,61,72,74] |
| Human Contact | How the AI technology will affect the patient-clinician encounter, especially its emotional and humane sides. | [19,26,51,70,74,75] |
| General AI Attitude | The effect of one's general attitude to digital technology adoption. | [51,71,72,75,76] |
| Awareness of Technology | Awareness about technology use and education about it. | [19,22,26,51,58,71] |
| Effect on Healthcare System Use | Effect of AI technology on one's access to healthcare services. | [26,51] |
| Data Destination | General concerns about the data privacy. | [70,74] |
| Usability | Ease of AI technology use. | [7,14,71–73] |
| Explainability | Explainability of the AI technology. | [16,43,70] |
| Accountability | Clear responsibility for the use of the AI technology in care. | [70] |
| Healthcare System Effects | Effect of the AI technology on the fairness and costs of the healthcare system. | [70] |
| Trust | Trust placed in the technology. | [22,72–75] |

**References**

1. Page MJ, McKenzie JE, Bossuyt PM, Boutron I, Hoffmann TC, Mulrow CD, Shamseer L, Tetzlaff JM, Akl EA, Brennan SE, Chou R, Glanville J, Grimshaw JM, Hróbjartsson A, Lalu MM, Li T, Loder EW, Mayo-Wilson E, McDonald S, McGuinness LA, Stewart LA, Thomas J, Tricco AC, Welch VA, Whiting P, Moher D. The PRISMA 2020 statement: an updated guideline for reporting systematic reviews. BMJ British Medical Journal Publishing Group; 2021 Mar 29;372:n71. PMID:33782057

2. Page MJ, Moher D, Bossuyt PM, Boutron I, Hoffmann TC, Mulrow CD, Shamseer L, Tetzlaff JM, Akl EA, Brennan SE, Chou R, Glanville J, Grimshaw JM, Hróbjartsson A, Lalu MM, Li T, Loder EW, Mayo-Wilson E, McDonald S, McGuinness LA, Stewart LA, Thomas J, Tricco AC, Welch VA, Whiting P, McKenzie JE. PRISMA 2020 explanation and elaboration: updated guidance and exemplars for reporting systematic reviews. BMJ British Medical Journal Publishing Group; 2021 Mar 29;372:n160. PMID:33781993

3. Zhai H, Yang X, Xue J, Lavender C, Ye T, Li J-B, Xu L, Lin L, Cao W, Sun Y. Radiation Oncologists’ Perceptions of Adopting an Artificial Intelligence–Assisted Contouring Technology: Model Development and Questionnaire Study. J Med Internet Res 2021 Sep 30;23(9):e27122. doi: 10.2196/27122

4. Thakkar B, Bharathi SV. Medical Specialists’ Perception About Adoption of Artificial Intelligence in the Healthcare Sector. CARDIOMETRY 2023 Feb 14;(25):426–434. doi: 10.18137/cardiometry.2022.25.426434

5. Cheng M, Li X, Xu J. Promoting Healthcare Workers’ Adoption Intention of Artificial-Intelligence-Assisted Diagnosis and Treatment: The Chain Mediation of Social Influence and Human–Computer Trust. Int J Environ Res Public Health 2022 Oct 15;19(20):13311. doi: 10.3390/ijerph192013311

6. Prakash AV, Das S. Medical practitioner’s adoption of intelligent clinical diagnostic decision support systems: A mixed-methods study. Inf Manage 2021 Nov;58(7):103524. doi: 10.1016/j.im.2021.103524

7. Kleine A-K, Kokje E, Lermer E, Gaube S. Attitudes Toward the Adoption of 2 Artificial Intelligence–Enabled Mental Health Tools Among Prospective Psychotherapists: Cross-sectional Study. JMIR Hum Factors 2023 Jul 12;10:e46859. doi: 10.2196/46859

8. Morrison K. Artificial intelligence and the NHS: a qualitative exploration of the factors influencing adoption. Future Healthc J 2021 Nov;8(3):e648–e654. doi: 10.7861/fhj.2020-0258

9. Zheng L, Ohde JW, Overgaard SM, Brereton TA, Jose KA, Wi C, Peterson KJ, Juhn YJ. User-Centered Design to Develop and Implement an ML-Based Asthma Management Tool Abstract. medRxiv 2022; doi: 10.1101/2022.12.28.22282986__;!!PDiH4ENfjr2_Jw!Gv6rYjE3iIsdhIIXKdK46iiQNHmIB6CVs3Er9ti4GOQUQsZlPABLL8R7-KijVSoR78sXM65Ta-q8E52ACKAWfacANfJV0dtnj-GPY0kU2k57mw$

10. Watson J, Hutyra CA, Clancy SM, Chandiramani A, Bedoya A, Ilangovan K, Nderitu N, Poon EG. Overcoming barriers to the adoption and implementation of predictive modeling and machine learning in clinical care: what can we learn from US academic medical centers? JAMIA Open 2020 Jul 1;3(2):167–172. doi: 10.1093/jamiaopen/ooz046

11. Yarborough BJH, Stumbo SP, Schneider J, Richards JE, Hooker SA, Rossom R. Clinical implementation of suicide risk prediction models in healthcare: a qualitative study. BMC Psychiatry 2022 Dec 14;22(1):789. doi: 10.1186/s12888-022-04400-5

12. Weinert L, Müller J, Svensson L, Heinze O. Perspective of Information Technology Decision Makers on Factors Influencing Adoption and Implementation of Artificial Intelligence Technologies in 40 German Hospitals: Descriptive Analysis. JMIR Med Inform 2022 Jun 15;10(6):e34678. doi: 10.2196/34678

13. Victor Mugabe K. Barriers and facilitators to the adoption of artificial intelligence in radiation oncology: A New Zealand study. Tech Innov Patient Support Radiat Oncol 2021 Jun;18:16–21. doi: 10.1016/j.tipsro.2021.03.004

14. Tricco AC, Hezam A, Parker A, Nincic V, Harris C, Fennelly O, Thomas SM, Ghassemi M, McGowan J, Paprica PA, Straus SE. Implemented machine learning tools to inform decision-making for patient care in hospital settings: a scoping review. BMJ Open 2023 Feb;13(2):e065845. doi: 10.1136/bmjopen-2022-065845

15. Petitgand C, Motulsky A, Denis JL, Regis C. Investigating the Barriers to Physician Adoption of an Artificial Intelligence- Based Decision Support System in Emergency Care: An Interpretative Qualitative Study. Stud Health Technol Inform 2020;270:1001–1005. doi: 10.3233/SHTI200312__;!!PDiH4ENfjr2_Jw!Gv6rYjE3iIsdhIIXKdK46iiQNHmIB6CVs3Er9ti4GOQUQsZlPABLL8R7-KijVSoR78sXM65Ta-q8E52ACKAWfacANfJV0dtnj-GPY0kw8L-mIQ$

16. Lokaj B, Pugliese M-T, Kinkel K, Lovis C, Schmid J. Barriers and facilitators of artificial intelligence conception and implementation for breast imaging diagnosis in clinical practice: a scoping review. Eur Radiol 2023 Sep 2; doi: 10.1007/s00330-023-10181-6

17. Chomutare T, Tejedor M, Svenning TO, Marco-Ruiz L, Tayefi M, Lind K, Godtliebsen F, Moen A, Ismail L, Makhlysheva A, Ngo PD. Artificial Intelligence Implementation in Healthcare: A Theory-Based Scoping Review of Barriers and Facilitators. Int J Environ Res Public Health 2022 Dec 6;19(23):16359. doi: 10.3390/ijerph192316359

18. Alexander N, Aftandilian C, Guo LL, Plenert E, Posada J, Fries J, Fleming S, Johnson A, Shah N, Sung L. Perspective Toward Machine Learning Implementation in Pediatric Medicine: Mixed Methods Study. JMIR Med Inform 2022 Nov 17;10(11):e40039. doi: 10.2196/40039

19. Van Der Vegt AH, Scott IA, Dermawan K, Schnetler RJ, Kalke VR, Lane PJ. Deployment of machine learning algorithms to predict sepsis: systematic review and application of the SALIENT clinical AI implementation framework. J Am Med Inform Assoc 2023 Jun 20;30(7):1349–1361. doi: 10.1093/jamia/ocad075

20. Mosch LK, Poncette A-S, Spies C, Weber-Carstens S, Schieler M, Krampe H, Balzer F. Creation of an Evidence-Based Implementation Framework for Digital Health Technology in the Intensive Care Unit: Qualitative Study. JMIR Form Res 2022 Apr 8;6(4):e22866. doi: 10.2196/22866

21. Dastjerdi M, Keramati A, Keramati N. A novel framework for investigating organizational adoption of AI-integrated CRM systems in the healthcare sector; using a hybrid fuzzy decision-making approach. Telemat Inform Rep 2023 Sep;11:100078. doi: 10.1016/j.teler.2023.100078

22. Abujaber AA, Nashwan AJ, Fadlalla A. Enabling the adoption of machine learning in clinical decision support: A Total Interpretive Structural Modeling Approach. Inform Med Unlocked 2022;33:101090. doi: 10.1016/j.imu.2022.101090

23. Baxter SL, Bass JS, Sitapati AM. Barriers to Implementing an Artificial Intelligence Model for Unplanned Readmissions. ACI Open 2020 Jul;04(02):e108–e113. doi: 10.1055/s-0040-1716748

24. Bentley KH, Zuromski KL, Fortgang RG, Madsen EM, Kessler D, Lee H, Nock MK, Reis BY, Castro VM, Smoller JW. Implementing Machine Learning Models for Suicide Risk Prediction in Clinical Practice: Focus Group Study With Hospital Providers. JMIR Form Res 2022 Mar 11;6(3):e30946. doi: 10.2196/30946

25. Burgess ER, Jankovic I, Austin M, Cai N, Kapuścińska A, Currie S, Overhage JM, Poole ES, Kaye J. Healthcare AI Treatment Decision Support: Design Principles to Enhance Clinician Adoption and Trust. Proc 2023 CHI Conf Hum Factors Comput Syst Hamburg Germany: ACM; 2023. p. 1–19. doi: 10.1145/3544548.3581251

26. Hogg HDJ, Al-Zubaidy M, Technology Enhanced Macular Services Study Reference Group, Talks J, Denniston AK, Kelly CJ, Malawana J, Papoutsi C, Teare MD, Keane PA, Beyer FR, Maniatopoulos G. Stakeholder Perspectives of Clinical Artificial Intelligence Implementation: Systematic Review of Qualitative Evidence. J Med Internet Res 2023 Jan 10;25:e39742. doi: 10.2196/39742

27. Wewetzer L, Held LA, Goetz K, Steinhäuser J. Determinants of the implementation of artificial intelligence-based screening for diabetic retinopathy—a cross-sectional study with general practitioners in Germany. Digit Health 2023 Jan;9:205520762311766. doi: 10.1177/20552076231176644

28. Van Cauwenberge D, Van Biesen W, Decruyenaere J, Leune T, Sterckx S. “Many roads lead to Rome and the Artificial Intelligence only shows me one road”: an interview study on physician attitudes regarding the implementation of computerised clinical decision support systems. BMC Med Ethics 2022 Dec;23(1):50. doi: 10.1186/s12910-022-00787-8

29. Held LA, Wewetzer L, Steinhäuser J. Determinants of the implementation of an artificial intelligence-supported device for the screening of diabetic retinopathy in primary care – a qualitative study. Health Informatics J 2022 Jul;28(3):146045822211128. doi: 10.1177/14604582221112816

30. Chen JS, Lin MC, Yiu G, Thorne C, Kulasa K, Stewart J, Nudleman E, Freeby M, Han MA, Baxter SL. Barriers to Implementation of Teleretinal Diabetic Retinopathy Screening Programs Across the University of California. Telemed E-Health 2023 Dec 1;29(12):1810–1818. doi: 10.1089/tmj.2022.0489

31. Al Badi FK, Alhosani KA, Jabeen F, Stachowicz-Stanusch A, Shehzad N, Amann W. Challenges of AI Adoption in the UAE Healthcare. Vis J Bus Perspect 2022 Jun;26(2):193–207. doi: 10.1177/0972262920988398

32. Yang J, Luo B, Zhao C, Zhang H. Artificial intelligence healthcare service resources adoption by medical institutions based on TOE framework. Digit Health 2022 Jan;8:205520762211260. doi: 10.1177/20552076221126034

33. Bergquist M, Rolandsson B, Gryska E, Laesser M, Hoefling N, Heckemann R, Schneiderman JF, Björkman-Burtscher IM. Trust and stakeholder perspectives on the implementation of AI tools in clinical radiology. Eur Radiol 2023 Jul 28;34(1):338–347. doi: 10.1007/s00330-023-09967-5

34. D’Hondt E, Ashby TJ, Chakroun I, Koninckx T, Wuyts R. Identifying and evaluating barriers for the implementation of machine learning in the intensive care unit. Commun Med 2022;2(1):162. doi: 10.1038/s43856-022-00225-1__;!!PDiH4ENfjr2_Jw!Gv6rYjE3iIsdhIIXKdK46iiQNHmIB6CVs3Er9ti4GOQUQsZlPABLL8R7-KijVSoR78sXM65Ta-q8E52ACKAWfacANfJV0dtnj-GPY0nfwrNejw$

35. Darcel K, Upshaw T, Craig-Neil A, Macklin J, Steele Gray C, Chan TCY, Gibson J, Pinto AD. Implementing artificial intelligence in Canadian primary care: Barriers and strategies identified through a national deliberative dialogue. Mohammadzadeh A, editor. PLOS ONE 2023 Feb 27;18(2):e0281733. doi: 10.1371/journal.pone.0281733

36. Fujimori R, Liu K, Soeno S, Naraba H, Ogura K, Hara K, Sonoo T, Ogura T, Nakamura K, Goto T. Acceptance, Barriers, and Facilitators to Implementing Artificial Intelligence–Based Decision Support Systems in Emergency Departments: Quantitative and Qualitative Evaluation. JMIR Form Res 2022 Jun 13;6(6):e36501. doi: 10.2196/36501

37. Joshi M, Mecklai K, Rozenblum R, Samal L. Implementation approaches and barriers for rule-based and machine learning-based sepsis risk prediction tools: a qualitative study. JAMIA Open 2022 Apr 6;5(2):ooac022. doi: 10.1093/jamiaopen/ooac022

38. Gavriilidis GI, Dimitriadis VK, Jaulent M-C, Natsiavas P. Identifying Actionability as a Key Factor for the Adoption of ‘Intelligent’ Systems for Drug Safety: Lessons Learned from a User-Centred Design Approach. Drug Saf 2021 Nov;44(11):1165–1178. doi: 10.1007/s40264-021-01103-w

39. Haugsten ER, Vestergaard T, Trettin B. Experiences Regarding Use and Implementation of Artificial Intelligence–Supported Follow-Up of Atypical Moles at a Dermatological Outpatient Clinic: Qualitative Study. JMIR Dermatol 2023 Jun 23;6:e44913. doi: 10.2196/44913

40. Iheme OC, Nattress D, Macht B. Artificial Intelligence-Based Medical Device Technologies Implementation Strategies in the Nigerian Health Care Industry. Walden University; 2023. Available from: https://urldefense.com/v3/__https://dl.acm.org/doi/book/10.5555/AAI30309399__;!!PDiH4ENfjr2_Jw!Gv6rYjE3iIsdhIIXKdK46iiQNHmIB6CVs3Er9ti4GOQUQsZlPABLL8R7-KijVSoR78sXM65Ta-q8E52ACKAWfacANfJV0dtnj-GPY0nzQNSXDQ$ [dl[.]acm[.]org]

41. Ji M, Chen X, Genchev GZ, Wei M, Yu G. Status of AI-Enabled Clinical Decision Support Systems Implementations in China. Methods Inf Med 2021 Dec;60(05/06):123–132. doi: 10.1055/s-0041-1736461

42. Labrague LJ, Aguilar-Rosales R, Yboa BC, Sabio JB. Factors influencing student nurses’ readiness to adopt artificial intelligence (AI) in their studies and their perceived barriers to accessing AI technology: A cross-sectional study. Nurse Educ Today 2023 Nov;130:105945. doi: 10.1016/j.nedt.2023.105945

43. Mlodzinski E, Wardi G, Viglione C, Nemati S, Crotty Alexander L, Malhotra A. Assessing Barriers to Implementation of Machine Learning and Artificial Intelligence–Based Tools in Critical Care: Web-Based Survey Study. JMIR Perioper Med 2023 Jan 27;6:e41056. doi: 10.2196/41056

44. Sax DR, Sturmer LR, Mark DG, Rana JS, Reed ME. Barriers and Opportunities Regarding Implementation of a Machine Learning-Based Acute Heart Failure Risk Stratification Tool in the Emergency Department. Diagnostics 2022 Oct 11;12(10):2463. doi: 10.3390/diagnostics12102463

45. Manetti S, Cumetti M, De Benedictis A, Lettieri E. Adoption of novel biomarker test parameters with machine learning‐based algorithms for the early detection of sepsis in hospital practice. J Nurs Manag 2022 Nov;30(8):3754–3764. doi: 10.1111/jonm.13807

46. Nair M, Andersson J, Nygren JM, Lundgren LE. Barriers and Enablers for Implementation of an Artificial Intelligence–Based Decision Support Tool to Reduce the Risk of Readmission of Patients With Heart Failure: Stakeholder Interviews. JMIR Form Res 2023 Aug 23;7:e47335. doi: 10.2196/47335

47. Misro A, Mehta A, Whittington P, Dogan H, Mishra N, Kadoglou N, Theivacumar S. Analysis of Innovation Adoption Challenges and Opportunities Surrounding Clinical Decision Support Systems (CDSS) in India. medRxiv 2023; doi: 10.1101/2023.04.02.23288046__;!!PDiH4ENfjr2_Jw!Gv6rYjE3iIsdhIIXKdK46iiQNHmIB6CVs3Er9ti4GOQUQsZlPABLL8R7-KijVSoR78sXM65Ta-q8E52ACKAWfacANfJV0dtnj-GPY0lKgODeUw$

48. Ramirez E, Hinga B, Foulk S. Artificial Intelligence in Medical Dosimetry: A Quantitative Analysis of Artificial Intelligence Adoption Among Medical Dosimetrists. University of Southern California; 2022. Available from: https://urldefense.com/v3/__https://dl.acm.org/doi/book/10.5555/AAI29255687__;!!PDiH4ENfjr2_Jw!Gv6rYjE3iIsdhIIXKdK46iiQNHmIB6CVs3Er9ti4GOQUQsZlPABLL8R7-KijVSoR78sXM65Ta-q8E52ACKAWfacANfJV0dtnj-GPY0lkhchEfA$ [dl[.]acm[.]org]

49. Romero-Brufau S, Wyatt KD, Boyum P, Mickelson M, Moore M, Cognetta-Rieke C. A lesson in implementation: A pre-post study of providers’ experience with artificial intelligence-based clinical decision support. Int J Med Inf 2020 May;137:104072. doi: 10.1016/j.ijmedinf.2019.104072

50. Sangers TE, Wakkee M, Moolenburgh FJ, Nijsten T, Lugtenberg M. Towards successful implementation of artificial intelligence in skin cancer care: a qualitative study exploring the views of dermatologists and general practitioners. Arch Dermatol Res 2022 Dec 7; doi: 10.1007/s00403-022-02492-3

51. Steerling E, Siira E, Nilsen P, Svedberg P, Nygren J. Implementing AI in healthcare—the relevance of trust: a scoping review. Front Health Serv 2023 Aug 24;3:1211150. doi: 10.3389/frhs.2023.1211150

52. Schouten B, Schinkel M, Boerman AW, Van Pijkeren P, Thodé M, Van Beneden M, Nannan Panday R, De Jonge R, Wiersinga WJ, Nanayakkara PWB. Implementing artificial intelligence in clinical practice: a mixed-method study of barriers and facilitators. J Med Artif Intell 2022 Dec;5:12–12. doi: 10.21037/jmai-22-71

53. Strohm L, Hehakaya C, Ranschaert ER, Boon WPC, Moors EHM. Implementation of artificial intelligence (AI) applications in radiology: hindering and facilitating factors. Eur Radiol 2020 Oct;30(10):5525–5532. doi: 10.1007/s00330-020-06946-y

54. Sibley MH, Bickman L, Atkins D, Tanana M, Coxe S, Ortiz M, Martin P, King J, Monroy JM, Ponce T, Cheng J, Pace B, Zhao X, Chawla V, Page TF. Developing an Implementation Model for ADHD Intervention in Community Clinics: Leveraging Artificial Intelligence and Digital Technology. Cogn Behav Pract 2023 Mar;S1077722923000196. doi: 10.1016/j.cbpra.2023.02.001

55. Joshi S, Sharma M, Das RP, Rosak-Szyrocka J, Żywiołek J, Muduli K, Prasad M. Modeling Conceptual Framework for Implementing Barriers of AI in Public Healthcare for Improving Operational Excellence: Experiences from Developing Countries. Sustainability 2022 Sep 18;14(18):11698. doi: 10.3390/su141811698

56. Dumbach P, Liu R, Jalowski M, Eskofier BM. The adoption of artificial intelligence in SMEs - A cross-national comparison in German and Chinese healthcare. 2021. p. 84–98. Available from: https://urldefense.com/v3/__https://www.scopus.com/inward/record.uri?eid=2-s2.0-85118858808&partnerID=40&md5=589f414350f260faddec83dbac177795__;!!PDiH4ENfjr2_Jw!Gv6rYjE3iIsdhIIXKdK46iiQNHmIB6CVs3Er9ti4GOQUQsZlPABLL8R7-KijVSoR78sXM65Ta-q8E52ACKAWfacANfJV0dtnj-GPY0kNXqp7Sw$ [scopus[.]com]

57. Spitzer MD, Wong P, Baqai H. Assessing Factors Influencing Adoption Rates and Usability of AI Decision-Making Applications in Healthcare: A Generic Qualitative Inquiry. Capella University; 2021. Available from: https://urldefense.com/v3/__https://dl.acm.org/doi/book/10.5555/AAI28417666__;!!PDiH4ENfjr2_Jw!Gv6rYjE3iIsdhIIXKdK46iiQNHmIB6CVs3Er9ti4GOQUQsZlPABLL8R7-KijVSoR78sXM65Ta-q8E52ACKAWfacANfJV0dtnj-GPY0mUtZZ-yQ$ [dl[.]acm[.]org]

58. Chew HSJ, Achananuparp P. Perceptions and Needs of Artificial Intelligence in Health Care to Increase Adoption: Scoping Review. J Med Internet Res 2022 Jan 14;24(1):e32939. doi: 10.2196/32939

59. Petersson L, Larsson I, Nygren JM, Nilsen P, Neher M, Reed JE, Tyskbo D, Svedberg P. Challenges to implementing artificial intelligence in healthcare: a qualitative interview study with healthcare leaders in Sweden. BMC Health Serv Res 2022 Dec;22(1):850. doi: 10.1186/s12913-022-08215-8

60. Hindocha S, Zucker K, Jena R, Banfill K, Mackay K, Price G, Pudney D, Wang J, Taylor A. Artificial Intelligence for Radiotherapy Auto-Contouring: Current Use, Perceptions of and Barriers to Implementation. Clin Oncol 2023 Apr;35(4):219–226. doi: 10.1016/j.clon.2023.01.014

61. Kabukye JK, Namugga J, Mpamani CJ, Katumba A, Nakatumba-Nabende J, Nabuuma H, Musoke SS, Nankya E, Soomre E, Nakisige C, Orem J. Implementing Smartphone-Based Telemedicine for Cervical Cancer Screening in Uganda: Qualitative Study of Stakeholders’ Perceptions. J Med Internet Res 2023 Oct 2;25:e45132. doi: 10.2196/45132

62. Goldstein J, Weitzman D, Lemerond M, Jones A. Determinants for scalable adoption of autonomous AI in the detection of diabetic eye disease in diverse practice types: key best practices learned through collection of real-world data. Front Digit Health 2023 May 18;5:1004130. doi: 10.3389/fdgth.2023.1004130

63. Gillan C, Milne E, Harnett N, Purdie TG, Jaffray DA, Hodges B. Professional implications of introducing artificial intelligence in healthcare: an evaluation using radiation medicine as a testing ground. J Radiother Pract 2019 Mar;18(1):5–9. doi: 10.1017/S1460396918000468

64. Liu X, Barreto EF, Dong Y, Liu C, Gao X, Tootooni MS, Song X, Kashani KB. Discrepancy between perceptions and acceptance of clinical decision support Systems: implementation of artificial intelligence for vancomycin dosing. BMC Med Inform Decis Mak 2023 Aug 11;23(1):157. doi: 10.1186/s12911-023-02254-9

65. Singer SJ, Kellogg KC, Galper AB, Viola D. Enhancing the value to users of machine learning-based clinical decision support tools: A framework for iterative, collaborative development and implementation. Health Care Manage Rev 2022 Apr;47(2):E21–E31. doi: 10.1097/HMR.0000000000000324

66. Jordan M, Hauser J, Cota S, Li H, Wolf L. The Impact of Cultural Embeddedness on the Implementation of an Artificial Intelligence Program at Triage: A Qualitative Study. J Transcult Nurs 2023 Jan;34(1):32–39. doi: 10.1177/10436596221129226

67. Chen Y, Stavropoulou C, Narasinkan R, Baker A, Scarbrough H. Professionals’ responses to the introduction of AI innovations in radiology and their implications for future adoption: a qualitative study. BMC Health Serv Res 2021 Dec;21(1):813. doi: 10.1186/s12913-021-06861-y

68. Goncalves J, Faria BM, Reis LP, Carvalho V, Rocha A. Data mining and electronic devices applied to quality of life related to health data. 2015 10th Iber Conf Inf Syst Technol CISTI Aveiro, Portugal: IEEE; 2015. p. 1–4. doi: 10.1109/CISTI.2015.7170627

69. Brown LA, Benhamou K, May AM, Mu W, Berk R. Machine Learning Algorithms in Suicide Prevention: Clinician Interpretations as Barriers to Implementation. J Clin Psychiatry 2020 Apr 21;81(3). doi: 10.4088/JCP.19m12970

70. Isbanner S, O’Shaughnessy P, Steel D, Wilcock S, Carter S. The Adoption of Artificial Intelligence in Health Care and Social Services in Australia: Findings From a Methodologically Innovative National Survey of Values and Attitudes (the AVA-AI Study). J Med Internet Res 2022;24(8):e37611. doi: 10.2196/37611

71. Kutia S, Chauhdary SH, Iwendi C, Liu L, Yong W, Bashir AK. Socio-Technological Factors Affecting User’s Adoption of eHealth Functionalities: A Case Study of China and Ukraine eHealth Systems. IEEE Access 2019;7:90777–90788. doi: 10.1109/ACCESS.2019.2924584

72. Ye T, Xue J, He M, Gu J, Lin H, Xu B, Cheng Y. Psychosocial Factors Affecting Artificial Intelligence Adoption in Health Care in China: Cross-Sectional Study. J Med Internet Res 2019 Oct 17;21(10):e14316. doi: 10.2196/14316

73. Gupta O, Joshi P, Gupta A. Prioritizing the enablers for adoption of AI based health care wearables among elderly people. 2023 4th Int Conf Intell Eng Manag ICIEM London, United Kingdom: IEEE; 2023. p. 1–4. doi: 10.1109/ICIEM59379.2023.10165740

74. Mikkelsen JG, Sørensen NL, Merrild CH, Jensen MB, Thomsen JL. Patient perspectives on data sharing regarding implementing and using artificial intelligence in general practice – a qualitative study. BMC Health Serv Res 2023 Apr 4;23(1):335. doi: 10.1186/s12913-023-09324-8

75. Frank D-A, Elbæk CT, Børsting CK, Mitkidis P, Otterbring T, Borau S. Drivers and social implications of Artificial Intelligence adoption in healthcare during the COVID-19 pandemic. Guidi B, editor. PLOS ONE 2021 Nov 22;16(11):e0259928. doi: 10.1371/journal.pone.0259928

76. Armero W, Gray KJ, Fields KG, Cole NM, Bates DW, Kovacheva VP. A survey of pregnant patients’ perspectives on the implementation of artificial intelligence in clinical care. J Am Med Inform Assoc 2022 Dec 13;30(1):46–53. doi: 10.1093/jamia/ocac200
