# Supplementary material for: Barriers and Facilitators to Health Care AI Adoption Among Those Living in Wales and Working in Health Care in Wales: Online Survey
Source: J Med Internet Res. 2025 Dec 5;27:e81543. doi: 10.2196/81543 (PMC12717503; doi:10.2196/81543)
Supplement: Multimedia Appendix 2 [file jmir_v27i1e81543_app2.pdf]

Survey about using healthcare artificial intelligence (AI) technologies in Wales

We want to know what would encourage you to have artificial intelligence (AI) technologies used to support making healthcare decisions. We also want to know what would stop you from having artificial intelligence (AI) technologies used to support making healthcare decisions. We are interested in the opinions of:

- members of the public living in Wales who are at least 16 years old

- any registered healthcare staff working in Wales that make treatment/therapy decisions together with/for patients

What is Artificial Intelligence (AI)
Artificial intelligence (AI) technologies are a type of computer software that can perform activities which would ordinarily require some thought and effort for a person to complete. For example, look at patient information and decide what treatment they should get. NHS Wales collects a lot of information about patients' health and care, including patients' health related quality of life. Using this information, it is possible that such artificial intelligence (AI) technologies might be developed to help with caring for patients in Wales. Here is a possible example of how such an artificial intelligence (AI) technology could be used in practice:

Somebody has a painful hip. They go to their GP and later see a specialist surgeon. As part of their care they have several tests, such as an X-ray, and fill out a questionnaire which asks them about how the pain in the hip affects their day-to-day life, such as doing shopping or seeing friends. The artificial intelligence (AI) technology takes all this information together and suggests if it would be good for the patient to have surgery or not.

Why are we doing this research
The information we collect in this survey will help make sure that if such artificial intelligence (AI) technologies are designed and used, it will happen in a way that is acceptable to patients and healthcare staff. To do this, we will use the information we get from this survey to write reports, articles or give presentations.

We are asking for demographic information in order to make sure participants are an accurate representation of the population of Wales. The responses are anonymous unless you choose to provide your email address at the end of the questionnaire. We will not pass your e-mail address on to anyone else or share any identifiable information.

How long will it take
It will take about 14 minutes to complete this voluntary survey.

If you have any questions in relation to this survey, please contact michal.pruski@wales.nhs.uk

Data Privacy statement:

- Cardiff and Vale UHB is the data controller for any personal data that is collected.
- The lawful basis for processing data under the UK GDPR is Article 6(1)(e) and Article 9(2)(j)
- Data will be retained in line with the Records Management Code of Practice for Health and Care 2022
- For any complaints in relation to how your data has been handled, please contact Uhb.Dpo@wales.nhs.uk
- To find out more about how your information is processed, please see our privacy notice at https://cavuhb.nhs.wales/use-of-site/privacy-policy/

* Required

1. Please confirm that you have read and understood the information above *

◯ I confirm that I have read and understood information above, including the privacy statement

2. If you want to continue with this voluntary survey please select one of the options below: *

- ◯ I am a registered healthcare professional working in Wales and I make treatment/therapy decisions together with/for patients
- ◯ I live in Wales and I am at least 16 years old
- ◯ I am neither of these two

About you

These questions are collected so that we can check how representative the results of this survey are of the population of Wales and to see if we have surveyed people with different attitudes towards artificial intelligence (AI) technologies.

3. Within which County do you live in: *

- Blaenau Gwent
- Bridgend
- Caerphilly
- Cardiff
- Carmarthenshire
- Ceredigion
- Conwy
- Denbighshire
- Flintshire
- Gwynedd
- Isle of Anglesey
- Merthyr Tydfil
- Monmouthshire
- Neath Port Talbot
- Newport
- Pembrokeshire
- Powys
- Rhondda Cynon Taf
- Swansea
- Vale of Glamorgan
- Torfaen
- Wrexham
- Prefer not to say

4. Which of the following best describes your ethnic group: *

- ◯ Welsh/English/Scottish/Northern Irish/British
- ◯ Irish
- ◯ Gypsy or Irish Traveller
- ◯ Roma
- ◯ Any other White background
- ◯ White and Black Caribbean
- ◯ White and Black African
- ◯ White and Asian
- ◯ Any other Mixed/Multiple ethnic background
- ◯ Indian
- ◯ Pakistani
- ◯ Bangladeshi
- ◯ Chinese
- ◯ Any other Asian background
- ◯ Caribbean
- ◯ African background
- ◯ Any other Black, Black Welsh, Black British, Caribbean or African background
- ◯ Arab
- ◯ Other ethnic group
- ◯ Prefer not to say

5. What is your sex: *

- ◯ Male
- ◯ Female
- ◯ Prefer not to say

6. What is your age *

- ◯ 16-24
- ◯ 25-34
- ◯ 35-49
- ◯ 50-64
- ◯ 65-74
- ◯ 75 or over
- ◯ Prefer not to say

7. In general, I support/oppose the use of artificial intelligence (AI) technologies in healthcare *

- ◯ I support
- ◯ I oppose
- ◯ I do not know

Healthcare artificial intelligence (AI) technologies

Throughout this section think about what would make you trust a healthcare artificial intelligence (AI) technology. Here is an example that you can use to help you think about these questions:

Somebody has a painful hip. They go to their GP and later see a specialist surgeon. As part of their care they have several tests, such as an X-ray, and fill out a questionnaire which asks them about how the pain in the hip affects their day-to-day life, such as doing shopping or seeing friends. The artificial intelligence (AI) technology takes all this information together and suggests if it would be good for the patient to have surgery or not.

In some of the questions we will make assumptions about artificial intelligence (AI) technologies, for example that they might be beneficial or that they will make things happen faster. This is because we are only interested in artificial intelligence (AI) technologies that would make things better.

8. Do you think you should be able to refuse having an artificial intelligence (AI) technology used in your care even if we would know that this technology worked well at helping improve healthcare outcomes in your circumstances? *

- Yes
- No
- I do not know

9. Which of the following institutions would you trust most and trust least to develop a healthcare artificial intelligence (AI) technology that would be used in your care (move the sentences up and down so the top is the most trusted and the bottom is the least trusted). *

- Academia / Universities
- The Government / Civil Service
- The NHS
- Private companies

10. If an artificial intelligence (AI) technology was used to help care for you, would you be happy for your information to be used to improve the technology? *

- Yes
- No
- I do not know

11. Would you prefer a healthcare professional to have more input into the decision-making process or would you prefer the technology to be more automated and make a decision faster? *

- I would prefer the technology to make a faster decision
- I would prefer that the healthcare professional has more input into my care
- I do not know

12. In general, if the healthcare artificial intelligence (AI) technology made a mistake: *

- ◯ I would prefer it mistakenly recommended treatment
- ◯ I would prefer it to not recommend treatment that was needed
- ◯ I do not know

13. Please rank the following as to how likely they would be to convince you to have an artificial intelligence (AI) technology used in your care (move the sentences up and down so the top is the most likely and the bottom is the least likely): *

- The use of the technology will not replace me having an appointment with my healthcare professional (such as a doctor or nurse)
- The use of the technology will speed-up my care
- My healthcare professional (such as a doctor or nurse) can explain to me why the technology made a specific recommendation
- Being told that the technology will work for people from a similar background to me and with similar needs
- My healthcare professional (such as a doctor or nurse) has a choice to follow the technology’s recommendation and can make a different decision if they think another option is better

14. Please rank the following as to how likely they would be to stop you from having an artificial intelligence (AI) technology used in your care (move the sentences up and down so the top is the most likely and the bottom is the least likely): *

- People not involved in my care could see or use my information which was used by the technology
- Not being told how the technology’s recommendation will improve my care
- My healthcare professional (such as a doctor or nurse) does not trust the technology
- Using the technology distracts my healthcare professional (such as a doctor or nurse) from seeing the bigger picture of my needs
- Not being told about its use and then discovering about it later by accident, or being pressured to agree to its use

15. What qualities would an artificial intelligence (AI) technology need for you to trust it and find it acceptable in you care? Rank the importance of the following principles (move the sentences up and down so the top is the most important and the bottom is the least important): *

- The information used by the technology is kept **confidential**
- It is clear **who is responsible** for problems and mistakes caused by the technology
- It is clear **how the technology works** and was developed
- You are **viewed as a unique person** when the technology is being used
- The technology **avoids doing harm**
- The impact of the technology on the **natural environment** is considered
- The impact of the technology on the work and **employment of healthcare staff** is considered
- The technology **treats all people fairly** irrespective of their background
- You being free to **make your own decisions**
- The **technology is effective** at helping your health

Below, please list the three most important things that would make you worried about having artificial intelligence (AI) used in your care

16. Please state what would make you most worried about having artificial intelligence (AI) used in your care *

17. Please state another important thing that would make you worried about having artificial intelligence (AI) used in your care *

18. Please state another important thing that would make you worried about having artificial intelligence (AI) used in your care *

Below, please list the three most important things that would make you trust having artificial intelligence (AI) used in your care

19. Please state one thing that would most make you trust having artificial intelligence (AI) used in your care *

20. Please state one other important thing that would make you trust having artificial intelligence (AI) used in your care *

21. Please state one other important thing that would make you trust having artificial intelligence (AI) used in your care *

About you

These questions are collected so that we can check how representative the results of this survey are of the healthcare staff working Wales and to see if we have surveyed staff with different attitudes towards artificial intelligence (AI) technologies.

22. Please select your staff group: *

- ◯ Doctor/Dentist
- ◯ Nurse/Midwife
- ◯ Pharmacist
- ◯ AHP
- ◯ Healthcare Scientist
- ◯ Other

23. Please select the area that best describes your work *

- ◯ Diagnostics
- ◯ Therapeutics
- ◯ Acute Care
- ◯ Primary Care
- ◯ Community Care
- ◯ Mental Health
- ◯ Other secondary care

24. Which is the main patient group that you work with: *

- ◯ Adults
- ◯ Children
- ◯ Both

25. Within which County do you work in: *

- Blaenau Gwent
- Bridgend
- Caerphilly
- Cardiff
- Carmarthenshire
- Ceredigion
- Conwy
- Denbighshire
- Flintshire
- Gwynedd
- Isle of Anglesey
- Merthyr Tydfil
- Monmouthshire
- Neath Port Talbot
- Newport
- Pembrokeshire
- Powys
- Rhondda Cynon Taf
- Swansea
- Vale of Glamorgan
- Torfaen
- Wrexham
- Prefer not to say

26. Which of the following best describes your ethnic group: *

- ◯ Welsh/English/Scottish/Northern Irish/British
- ◯ Irish
- ◯ Gypsy or Irish Traveller
- ◯ Roma
- ◯ Any other White background
- ◯ White and Black Caribbean
- ◯ White and Black African
- ◯ White and Asian
- ◯ Any other Mixed/Multiple ethnic background
- ◯ Indian
- ◯ Pakistani
- ◯ Bangladeshi
- ◯ Chinese
- ◯ Any other Asian background
- ◯ Caribbean
- ◯ African background
- ◯ Any other Black, Black Welsh, Black British, Caribbean or African background
- ◯ Arab
- ◯ Other ethnic group
- ◯ Prefer not to say

27. What is your sex: *

- ◯ Male
- ◯ Female
- ◯ Prefer not to say

28. What is your age *

- ◯ 16-24
- ◯ 25-34
- ◯ 35-49
- ◯ 50-64
- ◯ 65-74
- ◯ 75 or over
- ◯ Prefer not to say

29. In general, I support/oppose the use of artificial intelligence (AI) technologies in healthcare *

- ◯ I support
- ◯ I oppose
- ◯ I do not know

Healthcare artificial intelligence (AI) technologies

Throughout this section think about what would make you trust a healthcare artificial intelligence (AI) technology. Here is an example that you can use to help you think about these questions:

Somebody has a painful hip. They go to their GP and later see a specialist surgeon. As part of their care they have several tests, such as an X-ray, and fill out a questionnaire which asks them about how the pain in the hip affects their day-to-day life, such as doing shopping or seeing friends. The artificial intelligence (AI) technology takes all this information together and suggests if it would be good for the patient to have surgery or not.

In some of the questions we will make assumptions about artificial intelligence (AI) technologies, for example that they might be beneficial or that they will make things happen faster. This is because we are only interested in artificial intelligence (AI) technologies that would make things better.

30. If an artificial intelligence (AI) technology was shown to work well in helping to improve patients' healthcare outcomes, should clinicians be able to object to using this technology in their clinical practice? *

- ◯ Yes
- ◯ No
- ◯ I do not know

31. Which of the following institutions would you trust most and trust least to develop a healthcare artificial intelligence (AI) technology that would be used in your clinical practice (move the sentences up and down so the top is the most trusted and the bottom is the least trusted). *

- The NHS
- Academia / Universities
- The Government / Civil Service
- Private companies

32. If an artificial intelligence (AI) technology was used in your clinical practice, would you support using information from your patients' care to improve the way the technology works? *

- ◯ Yes
- ◯ No
- ◯ I do not know

33. Would you prefer to have more input into the clinical decision-making process or would you prefer the artificial intelligence (AI) technology to be more automated and make a decision faster? *

- ◯ I would prefer the technology to make a faster decision
- ◯ I prefer to have more input into the care process
- ◯ I do not know

34. In general, if the healthcare artificial intelligence (AI) technology made a mistake: *

- ◯ I would prefer it mistakenly recommended treatment
- ◯ I would prefer it to not recommend treatment that was needed
- ◯ I do not know

35. Please rank the following in order of importance in being facilitators to you in using an artificial intelligence (AI) technology in the care of your patients (move the sentences up and down so the top is the most important and the bottom is the least important) *

- There is pre- and post-implementation (e.g. through monitoring) evidence for the AI's clinical effectiveness in your patient cohort
- There is national guidance for AI implementation, inclusive of clear criteria for legal liability when using AI in clinical practice
- There is transparency about what happens with the data collected by the AI and I agree with where it goes
- AI provides binary recommendations (e.g. recommends surgery or medical care) rather than statistics about the likelihood of patient satisfaction with each treatment option
- Well designed and conveniently delivered AI training

36. Please rank the following in order of importance of being a barrier to you in using an artificial intelligence (AI) technology in the care of your patients (move the sentences up and down so the top is the most important and the bottom is the least important) *

- My colleagues have a negative attitude towards AI
- AI decreasing clinicians' freedom to make decisions
- Lack of reassurance about the AI's performance in my patient groups (including patients from minority groups) and setting
- AI will distract me from attending to my patients' needs
- Lack of stakeholder (e.g. clinician and patient) input into the AI's development

37. What qualities would an artificial intelligence (AI) technology need for you to trust it and find it acceptable in the care of your patients. Rank the importance of the following principles (move the sentences up and down so the top is the most important and the bottom is the least important): *

- You being free to **make your own decisions**
- The impact of the technology on the work and **employment of healthcare staf**f is considered
- The information used by the technology is kept **confidential**
- The impact of the technology on the **natural environment** is considered
- The **technology is effective** in improving patient health
- Patients are **viewed as a unique persons** when the technology is being used
- The technology **treats all people fairly** irrespective of their background
- The technology **avoids doing harm**
- It is **clear how the technology works** and was developed
- It is clear **who is responsible** for problems and mistakes caused by the technology

Below, please list the three most important things that would make you hesitant about using artificial intelligence (AI) in your patients' care

38. Please state what would make you most hesitant about using artificial intelligence (AI) in your patients' care *

39. Please state another important thing that would make you hesitant about using artificial intelligence (AI) in your patients' care *

40. Please state another important thing that would make you hesitant about using artificial intelligence (AI) in your patients' care *

Below, please list one thing in each box that would reassure you about using artificial intelligence (AI) in your patients' care

41. Please state one thing that would reassure you most about using artificial intelligence (AI) in your patients' care *

42. Please state one other important thing that would reassure you about using artificial intelligence (AI) in your patients' care *

43. Please state one other important thing that would reassure you about using artificial intelligence (AI) in your patients' care *

44. The next phase of this project will involve focus groups and interviews. If you are happy to be contacted about participating in such a focus group or interview, please provide your name and email address *

- ◯ I do not want to be contacted
- ◯ I want to give my name and e-mail address

45. I want to be contacted in

- ◯ Welsh
- ◯ English

46. My name is

47. My e-mail address is

This content is neither created nor endorsed by Microsoft. The data you submit will be sent to the form owner.

Microsoft Forms
