# Supplementary material for: Barriers and Facilitators to Health Care AI Adoption Among Those Living in Wales and Working in Health Care in Wales: Online Survey
Source: J Med Internet Res. 2025 Dec 5;27:e81543. doi: 10.2196/81543 (PMC12717503; doi:10.2196/81543)
Supplement: Multimedia Appendix 3 [file jmir_v27i1e81543_app3.docx]

# **Additional tables and figures**

**Additional healthcare professional demographics information.**

| **Characteristic** | **% of Healthcare Professionals (n=130)** |
| --- | --- |
| *Staff Grouping* | |
| Doctor/Dentist | 24.6 (n=32) |
| Nurse/Midwife | 13.8 (n=18) |
| Pharmacist | 13.8 (n=18) |
| AHP | 16.9 (n=22) |
| Healthcare Scientist | 22.3 (n=29) |
| Other | 8.5 (n=11) |
| *Primary Area of Work* | |
| Diagnostics | 20.8 (n=27) |
| Therapeutics | 16.9 (n=22) |
| Acute Care | 15.4 (n=20) |
| Primary Care | 8.5 (n=11) |
| Community Care | 6.9 (n=9) |
| Mental Health | 2.3 (n=3) |
| Other Secondary Care | 29.2 (n=38) |
| *Primary Patient Group* | |
| Adults | 61.5 (n=80) |
| Children | 4.6 (n=6) |
| Both | 33.8 (n=44) |

Key demographics composition for those with self-reported positive, negative and uncertain attitudes to healthcare AI compared to the whole respondent population.


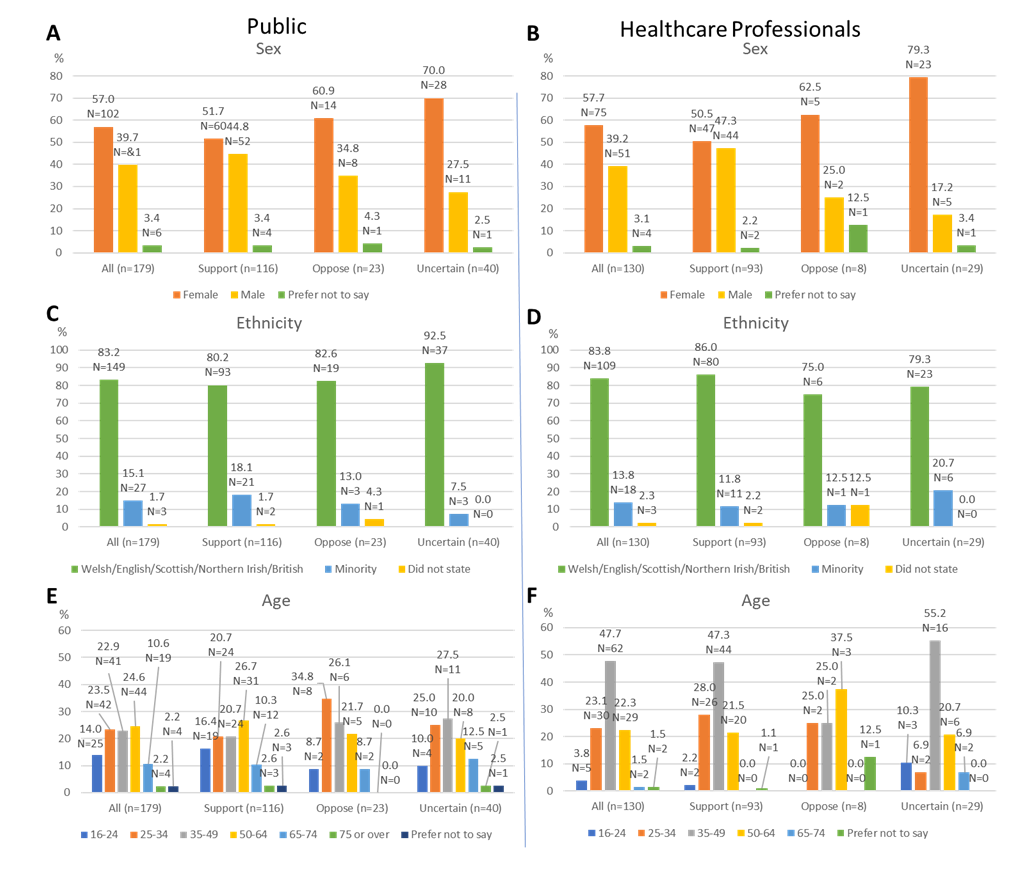


**Additional discussion on demographic data**

There are some limitations to both respondent groups’ reference data, beyond the fact that it is not fully-up to date. The NHS Wales staff data reflects all groupings employed in NHS Wales and not just registered professionals involved in treatment or therapy decisions and does not account for healthcare staff working in Wales, but outside of the NHS. Moreover, as highlighted in the legend of Table 1 in the main article, that the age categories did not match exactly those used in the NHS Wales statistics and are in general off-set by a year. It was not possible to use the same age categories among both respondent groups and match their available comparators without having to ask for exact age, which was not done to minimise privacy concerns. ONS categories were used as these are the generally recommended categories for survey use and the members of the public group was expected to be larger than the healthcare professional group.

Similarly, to minimise privacy concerns due to few responses from some counties, data was agglomerate into health board territories from county level data, since most healthcare services are delivered at health board level, with health board boundaries matching county agglomerate boundaries.[1] Moreover, such data presentation allows healthcare leaders to assess how representative the survey is of their populations, although patients might be treated in other health boards than their own, especially if they are accessing specialised services. No separate reference data was presented for healthcare staff, as additionally to the limitations regarding reference data which were mentioned at the beginning of this section, there are additional complexities to NHS Wales healthcare delivery model that would make the comparator data even less reliable. For example, several services are delivered via trusts rather than health boards, with such organisation, Digital Health and Care Wales and the specialist cancer trust Velindre, are based primarily in Cardiff, but are not part of the Cardiff and Vale University Health Board.

Healthcare professionals from all major staff groups responded to the survey (Supplementary File 4). We did not include comparator data as it is unclear for some of the groups what proportion of respondents from each group would be involved in treatment or therapy decisions. It is worth reiterating here, that this eligibility criterion might have been variously interpreted amongst respondents, and that some the respondents in this group have only a small involvement in such decisions, while some healthcare professionals who had only a small involvement in such decisions might have selected the other eligibility criterion. Nevertheless, we likely received a disproportionately high level of responses from the healthcare scientist staff group, due to extensive professional contacts among this cohort.

Lastly, though not important in the aggregated data, we got a larger than expected response rate from the ‘any other white background’ group due to one researcher being a fluent Polish speaker and being able to advertise the survey on social media groups for Polish people living in Wales.

**Mapping of the phrases used to describe the various ethics principles** to the two legibility criteria respondent groups to the principles themselves (questions 15 and 37 in the questionnaire; supplementary file 3).

| **Principle** | **Public** | **Healthcare Professionals** |
| --- | --- | --- |
| Dignity | You are **viewed as a unique person** when the technology is being used | Patients are **viewed as a unique persons** when the technology is being used |
| Confidentiality | The information used by the technology is kept **confidential** | The information used by the technology is kept **confidential** |
| Fairness | The technology **treats all people fairly** irrespective of their background | The technology **treats all people fairly** irrespective of their background |
| Responsibility | It is clear **who is responsible** for problems and mistakes caused by the technology | It is clear **who is responsible** for problems and mistakes caused by the technology |
| Autonomy | You being free to **make your own decisions** | You being free to **make your own decisions** |
| Solidarity | The impact of the technology on the work and **employment of healthcare staff** is considered | The impact of the technology on the work and **employment of healthcare staff** is considered |
| Environmental Impact | The impact of the technology on the **natural environment** is considered | The impact of the technology on the **natural environment** is considered |
| Nonmaleficence | The technology **avoids doing harm** | The technology **avoids doing harm** |
| Beneficence | The **technology is effective** at helping your health | The **technology is effective** in improving patient health |
| Transparency | It is clear **how the technology works** and was developed | It is **clear how the technology works** and was developed |

**Themes and sub-themes of the free-text answers**

Note that two barrier themes and one facilitator theme do not have sub-themes.

**
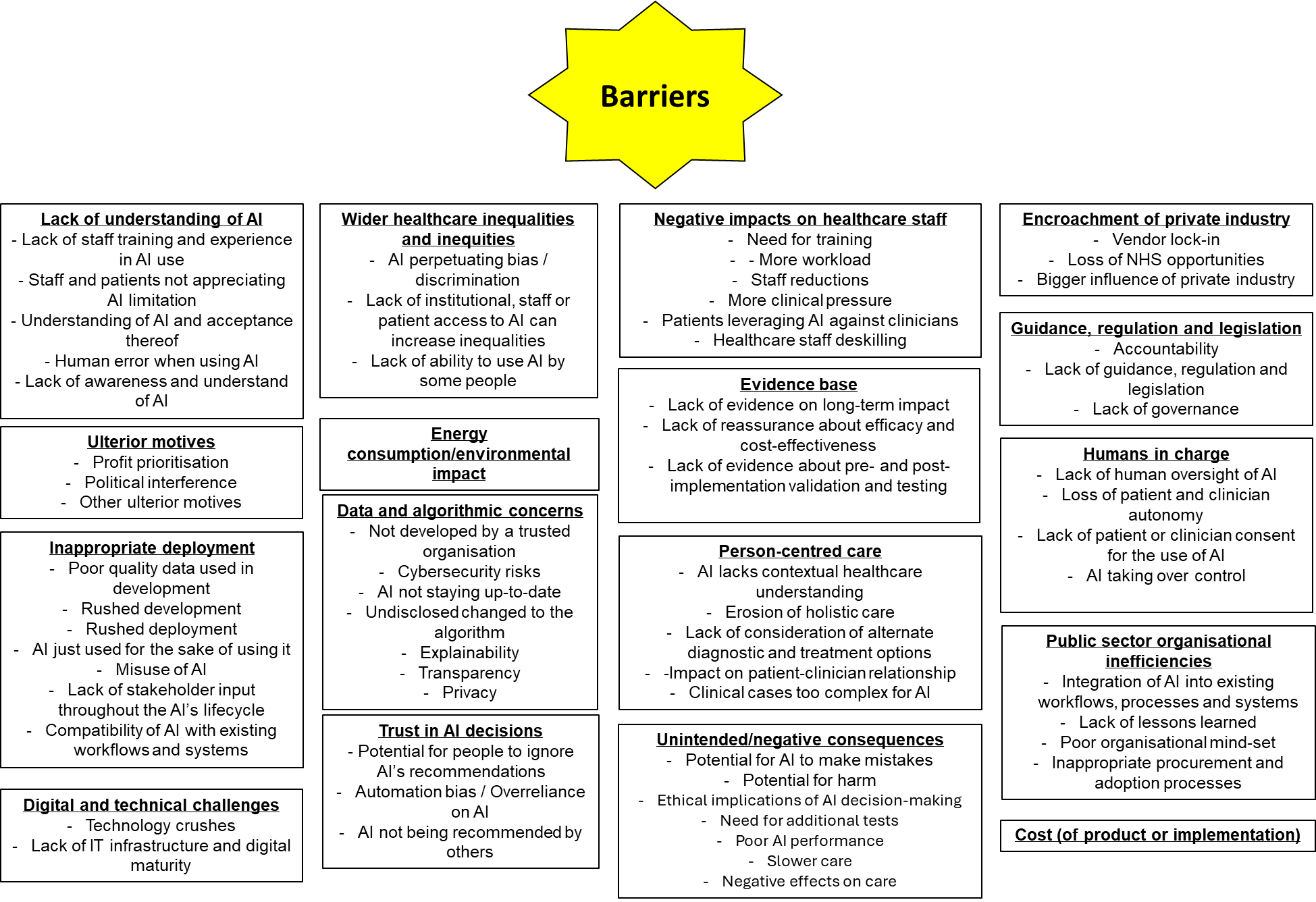
**

**
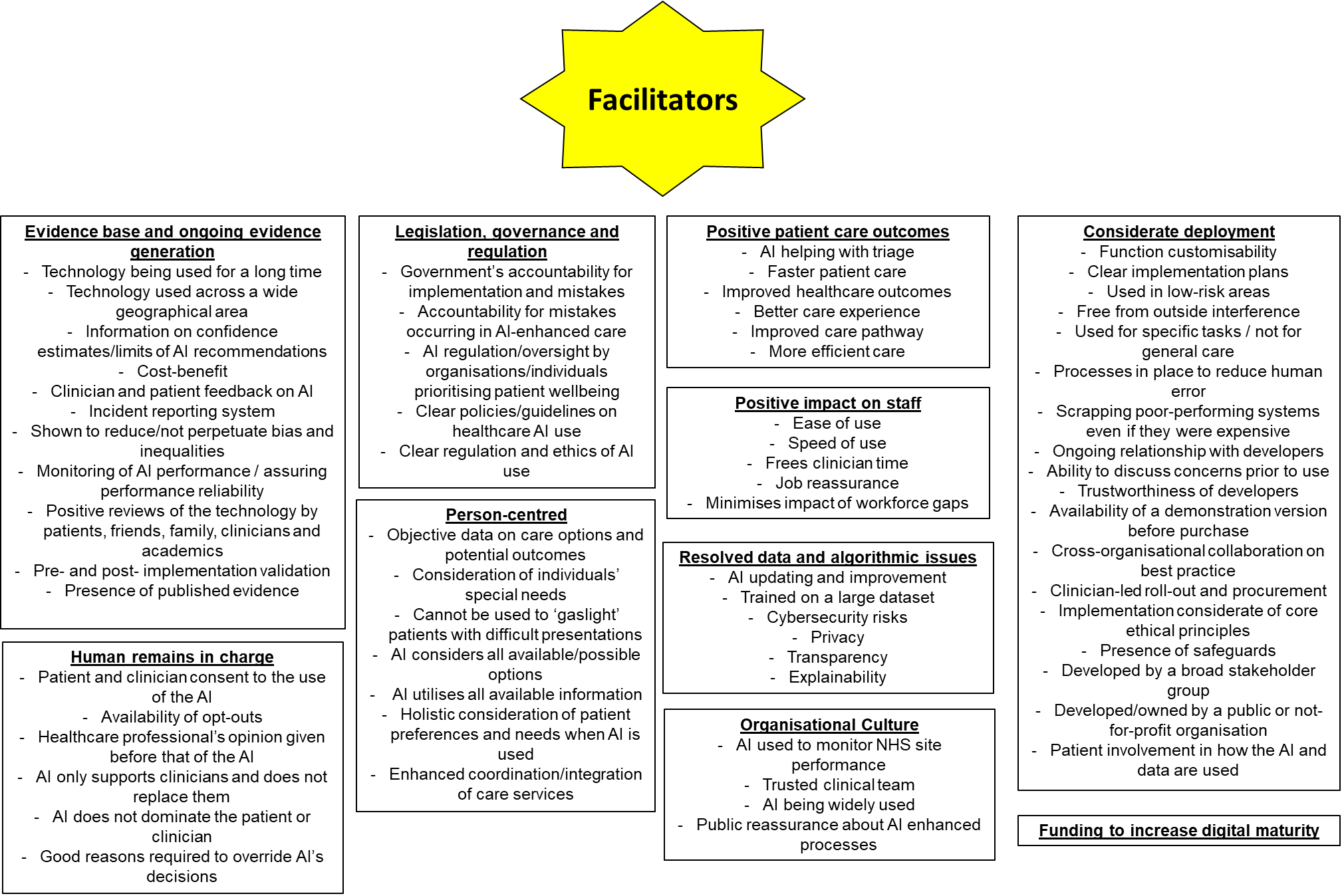
**

**Count of all the mentioned barrier themes to AI adoption stated by particpants and normalised to the number of items states in each group. N number indicates the number of counted items.**

**
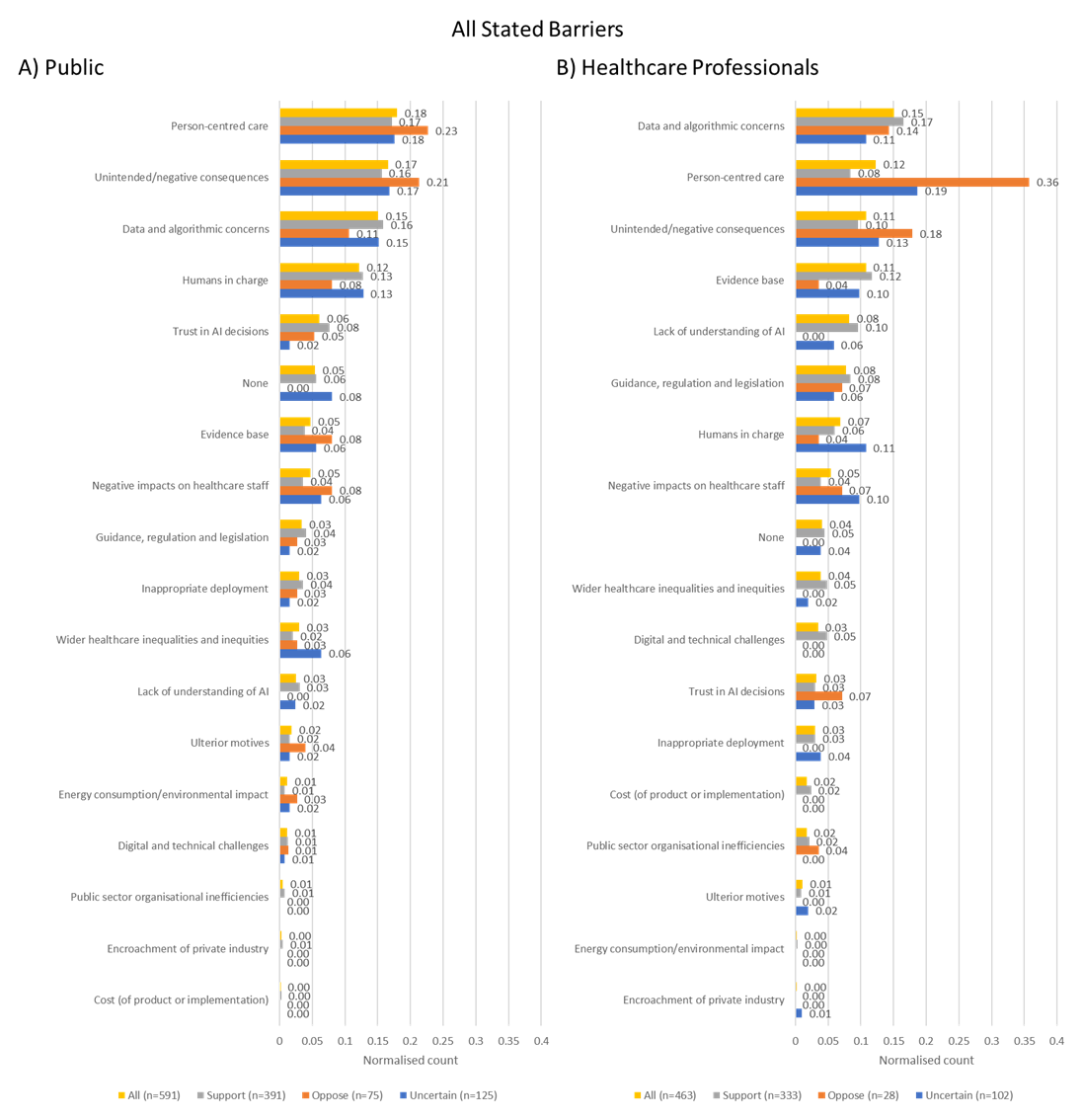
**

**Count of all the mentioned fascilitator themes to AI adoption stated by particpants and normalised to the number of items states in each group. N numnber indicates the number of counted items. Note that each theme received at least mention among all participants, but small numbers of mentios might not visusalise well on the graphs.**

**
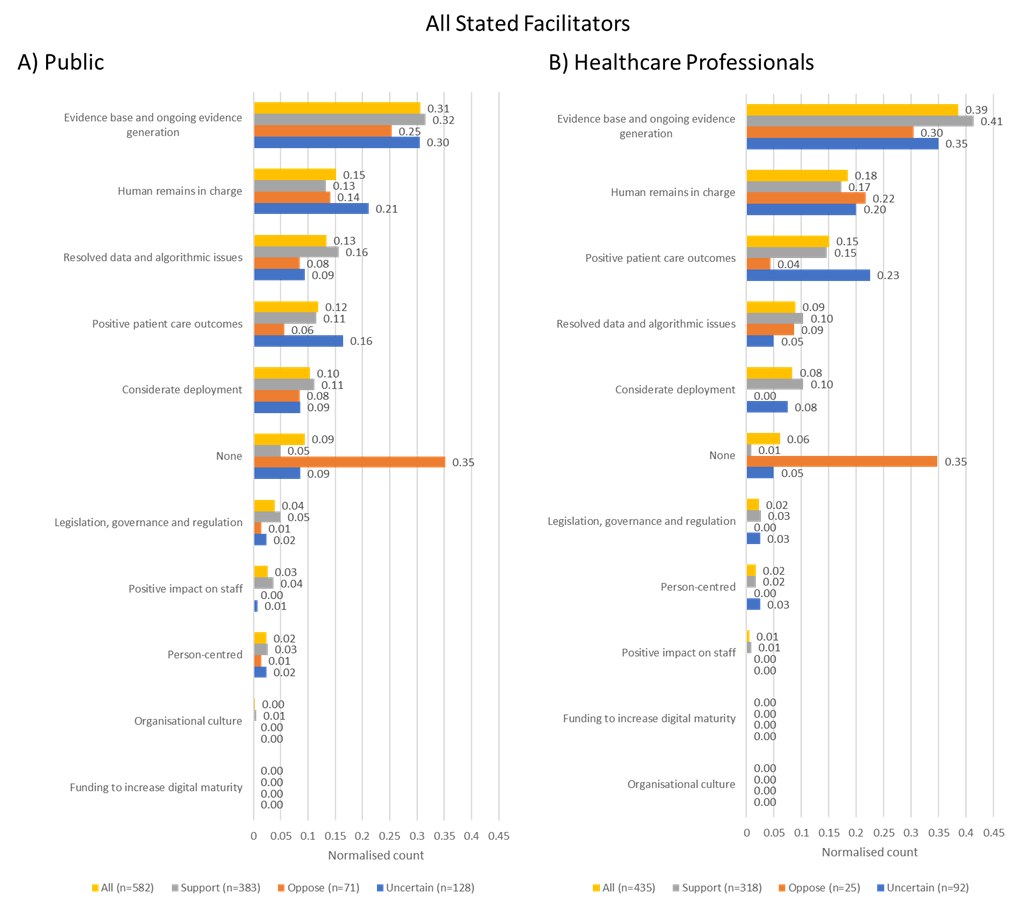
**

**References**

1. Welsh Government. NHS Wales health boards and trusts. GOVWALES. 2023. Available from: https://www.gov.wales/nhs-wales-health-boards-and-trusts [accessed May 5, 2025]
