# Supplementary figures and images for: Barriers and Facilitators to Health Care AI Adoption Among Those Living in Wales and Working in Health Care in Wales: Online Survey
Source: J Med Internet Res. 2025 Dec 5;27:e81543. doi: 10.2196/81543 (PMC12717503; doi:10.2196/81543)

**Google search statistics as of June 21, 2025 (<https://trends.google.com/trends/>)**

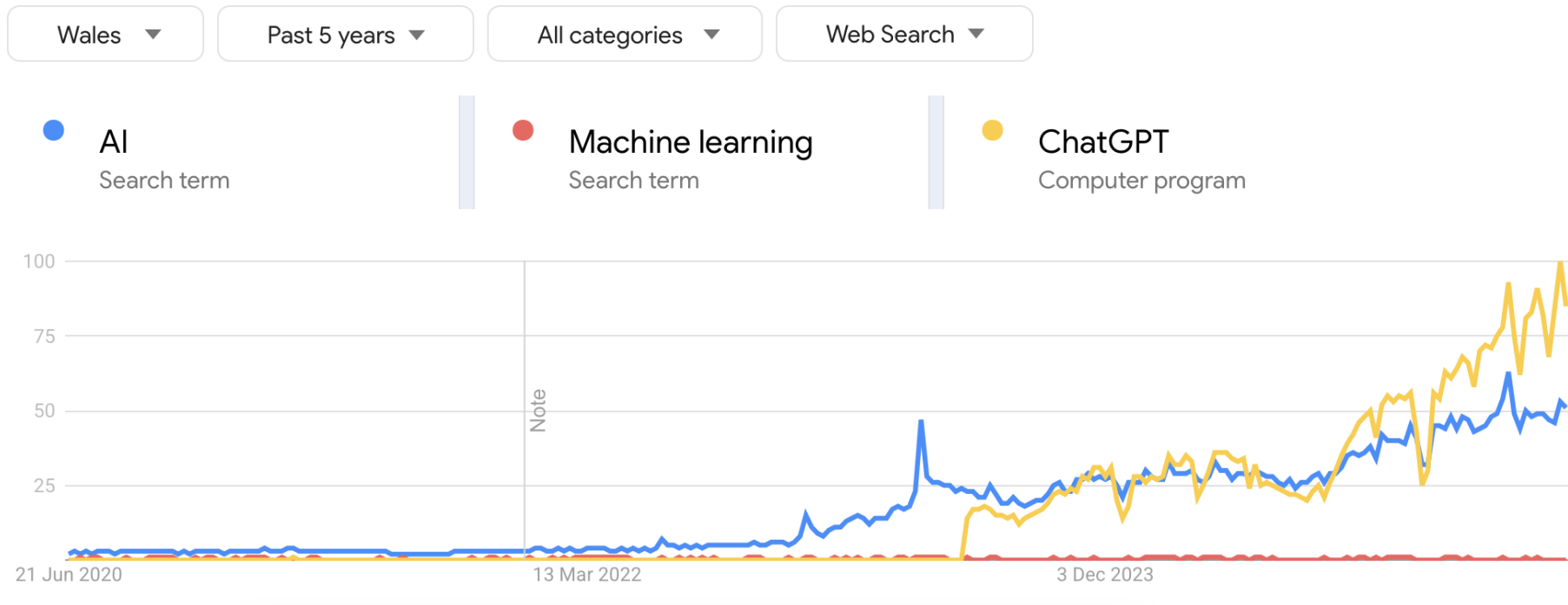

Supplement: Multimedia Appendix 4 [file jmir_v27i1e81543_app4.pdf]
